# Differences in the organization of interface residues tunes the stability of the SARS-CoV-2 spike-ACE2 complex

**DOI:** 10.3389/fmolb.2023.1205919

**Published:** 2023-06-27

**Authors:** Mattia Miotto, Lorenzo Di Rienzo, Greta Grassmann, Fausta Desantis, Gianluca Cidonio, Giorgio Gosti, Marco Leonetti, Giancarlo Ruocco, Edoardo Milanetti

**Affiliations:** ^1^ Center for Life Nano-& Neuro-Science, Istituto Italiano di Tecnologia, Rome, Italy; ^2^ Department of Biochemical Sciences “Alessandro Rossi Fanelli”, Sapienza University of Rome, Rome, Italy; ^3^ The Open University Affiliated Research Centre at Istituto Italiano di Tecnologia, Genova, Italy; ^4^ Soft and Living Matter Laboratory, Institute of Nanotechnology, Consiglio Nazionale delle Ricerche, Rome, Italy; ^5^ Department of Physics, Sapienza University of Rome, Rome, Italy

**Keywords:** SARS-C0V-2, complex stability, Zernike 2D, SARS-COV-2 variants, energetic interactions

## Abstract

The continuous emergence of novel variants represents one of the major problems in dealing with the SARS-CoV-2 virus. Indeed, also due to its prolonged circulation, more than ten variants of concern emerged, each time rapidly overgrowing the current viral version due to improved spreading features. As, up to now, all variants carry at least one mutation on the spike Receptor Binding Domain, the stability of the binding between the SARS-CoV-2 spike protein and the human ACE2 receptor seems one of the molecular determinants behind the viral spreading potential. In this framework, a better understanding of the interplay between spike mutations and complex stability can help to assess the impact of novel variants. Here, we characterize the peculiarities of the most representative variants of concern in terms of the molecular interactions taking place between the residues of the spike RBD and those of the ACE2 receptor. To do so, we performed molecular dynamics simulations of the RBD-ACE2 complexes of the seven variants of concern in comparison with a large set of complexes with different single mutations taking place on the RBD solvent-exposed residues and for which the experimental binding affinity was available. Analyzing the strength and spatial organization of the intermolecular interactions of the binding region residues, we found that (i) mutations producing an increase of the complex stability mainly rely on instaurating more favorable van der Waals optimization at the cost of Coulombic ones. In particular, (ii) an anti-correlation is observed between the shape and electrostatic complementarities of the binding regions. Finally, (iii) we showed that combining a set of dynamical descriptors is possible to estimate the outcome of point mutations on the complex binding region with a performance of 0.7. Overall, our results introduce a set of dynamical observables that can be rapidly evaluated to probe the effects of novel isolated variants or different molecular systems.

## Introduction

Severe Acute Respiratory Syndrome Coronavirus 2 (SARS-CoV-2) has been first observed in late 2019 in the Chinese region of Wuhan. From there, it rapidly spread all over the world, resulting in the Coronavirus Disease 2019 (COVID-19) global pandemic (Zhou et al., 2020; SalimKarim and de Oliveira, 2021). Despite the deployment of social distancing measures and a huge vaccination campaign, to date SARS-CoV-2 is still circulating and has caused, according to World Health Organization (WHO) on August 2022, over 600 million cases and 6 million deaths (Jonas et al., 2022).

At the molecular level, SARS-CoV-2 relies on the viral spike (S) glycoprotein to attach and enter the host cells. The spike protein has a homotrimeric structure that contacts receptors on the cell surface (Alejandra Tortorici and Veesler, 2019; Turoňová et al., 2020). In particular, attachment and entry processes are mediated by two distinct regions on the S protein: a region in the protein N-Terminal Domain (NTD) is apt to bind to sialoside molecules (Milanetti et al., 2021a), allowing for the initial attachment of the viral capsid to the cell surface, while another spike region, the Receptor Binding Domain (RBD), contacts its cellular receptor, Angiotensin-Converting Enzyme 2 (ACE2) (Yan et al., 2020; Cerutti et al., 2021).

Notably, this dual receptor mechanism has been previously observed in other coronaviruses. In fact, SARS-CoV-2 belongs to the family of beta coronaviruses and represents the third highly pathogenic coronavirus with a zoonotic origin that emerged in humans causing respiratory illness (Cui et al., 2019; Hu et al., 2021). Previous epidemics of SARS-CoV and MERS-CoV were registered in 2002–2004 and 2012 (Kumar et al., 2020). While SARS-CoV-2 shares the same entry receptor as SARS-CoV, the latter is unable to bind sialoside molecules, given the different conformation and length of the loops in the N-Terminal region. MERS-CoV, on the other hand, uses a different kind of entry receptor, dipeptidyl peptidase-4 (DPP4), but its NTD is able to establish avidic interactions with sialic acid moieties (Alejandra Tortorici and Veesler, 2019), which was first computationally predicted (Milanetti et al., 2021a) and then experimentally confirmed also for SARS-CoV-2 (Baker et al., 2020). Understanding the entry mechanisms of the virus through the molecular interactions with the receptors of the host cell is of crucial importance also to try to infer the possible consequences of mutations that take place both in the binding regions and in the more distant regions, which may have a long-range effect on the binding regions.

Similar to the other coronaviruses, mutations in the SARS-CoV-2 genetic code randomly occur in viral replication, where the ones that increase the fitness are preserved giving rise to new variants (Domingo and Holland, 1997; Duchene et al., 2020; Miotto and Monacelli, 2020; Portelli et al., 2020; Miotto et al., 2022a). For instance, concerning the original line of SARS-CoV-2, one of the first registered mutations was the amino acid substitution D614G in the S protein. Established in March 2020, this mutation allowed the spike RBD to assume a conformation more suitable for binding ACE2 and rapidly became dominant (Ralph, 2020; Trucchi et al., 2021; Zhang et al., 2021).

Indeed, RNA viruses are characterized by a low replication fidelity, which allows adaptation to different environments and evolutive pressure, in turn enabling them to escape the host immunity (Domingo and Holland, 1997; Mittal et al., 2022). In this scenario, during the spreading pandemic, several SARS-CoV-2 variants of concern (VOCs) have emerged. In particular, the alpha variant (lineage B.1.1.7), characterized by a mutation N501Y in its RBD (Harrington et al., 2021), was the first VOC detected during the COVID-19 pandemic, identified in November 2020 from a sample taken in September in the United Kingdom (Faria et al., 2021). It began to spread quickly by mid-December, around the same time as infections surged, being 40%–80% more transmissible than the wild-type (WT) SARS-CoV-2 (Chia et al., 2021; Lin et al., 2021). In December 2020 the beta variant (lineage B.1.351) was detected in South Africa (Tegally et al., 2020), even though phylogeographic analysis suggests this variant emerged much sooner, in July or August 2020 (Tegally et al., 2020). Nowadays, the WHO considers it to be no longer in circulation. It has been proposed that this variant is able to attach more easily to human cells because of three mutations in the RBD (Grabowski et al., 2021): N501Y, K417N, E484K. E484K and N501Y are included in the receptor-binding motif (RBM) of the RBD. In late 2020, other two VOCs, both descending from lineage B.1.617.2, were detected in India: the delta variant, carrying mutations L452R and T478K in the spike RBD (Adam, 2021), and the kappa variant, characterized by mutations L452R and E484K in that domain (Cherina et al., 2021).

In May 2021, the delta was declared quicker in its spread than both the original version of the virus and the alpha variant (Campbell et al., 2021). According to the WHO, in June 2021 this strain was becoming the dominant one globally, and by November 2021 it had spread to over 179 countries.

Also in December 2020, the first cases of the eta variant (lineage B.1.525) were detected in the UK and Nigeria (Liu et al., 2021). Eta is currently regarded as a variant under investigation, but pending further study, it may become a VOC (Liu et al., 2021). It presents in the RBD the mutation E484K.In January 2021 the gamma variant (descending from lineage B.1.1.28) was identified in Japan, in four people traveling from Brazil (Faria et al., 2021). Gamma has 17 amino acid substitutions, ten of which are in its spike protein, including three in the RBD: N501Y, E484K, K417T (Faria et al., 2021).The latest variant under study is omicron (lineage B.1.1.529), first reported in South Africa on 24 November 2021 (Gowrisankar et al., 2022) and, at the time of this study, the predominant variant in circulation, with a risk of reinfection higher than the other strains (Vitiello et al., 2022). Compared to any previous variant, omicron has more mutations, many of which are novel. It is characterized by 30 amino acid changes, three small deletions, and one small insertion in the spike protein compared with the original virus. Fifteen mutations are located in the RBD (Vitiello et al., 2022).

The prolonged circulation of the SARS-CoV-2 virus is favoring the emergence of novel variants. Thus, fast and specific methods to assess the impact of such variants are of great importance. In this respect, computational protocols able to operate from the genomic sequence would be ideal to cut off the delay between the identification of a novel strain and the clinical assessment of its spreading capabilities.

To make progress in this direction, here we focus on the experimental binding affinity between the SARS-CoV-2 spike protein and its human ACE2 receptor. We first carried out an analysis of the effect that single mutations on the spike RBD produce on the complex binding affinity. We then proposed a computational pipeline to probe the stabilizing/destabilizing role of mutations observed in VOCs with respect to a bunch of single-mutation variants. Our computational analysis, based on single mutations and the corresponding known binding affinity for the specific system, allows us to better elucidate the dynamic-structural properties of molecular complexes that are able to discriminate mutations on the basis of the effect on the binding affinity between the spike protein and the ACE2 receptor.

## Results and discussion

To investigate the effects of mutations on the stability of the SARS-CoV2 spike-ACE2 complex, we compared the WT complex of SARS-CoV-2 spike protein bound to human ACE2 receptor with 29 complexes obtained by computational mutation of some residues on the spike RBD and seven VOCs (alpha, beta, gamma, delta, eta, kappa and omicron). For each complex, we selected the ACE2 residues from 19 to 615 in complex with spike residues from 333 to 526, and we only considered the mutations in the spike RBD (including residues from 319 to 541), as listed in Tables 1, 2. The set of single-mutation variants, we selected, have experimental binding affinity data measured by Starr *et al.* (Starr et al., 2020) in a mutational scanning experiment with all possible single-mutation variants of the WT RBD. Computing the probability that a mutation at a certain position of the RBD sequence produces an increase of the affinity, one finds that such probabilities tend to be higher in regions that are in close proximity (e.g., closer than 10 Å) to the ACE2 receptor (see SI). Thus, we focused on the residues forming the binding region, i.e., residues 417, 455, 456, 475, 476, 484, 486, 487, 488, 489, 493, 494, 495, 496, 500, 501, 502. For each of these residues, we selected the mutation producing the highest and lowest binding affinity differences with respect to the WT complex. In addition, we included in the dataset both the single-mutation variants simulated in Miotto *et al.* (Miotto et al., 2022a) and those that appeared in the seven considered VOCs, i.e., alpha, beta, gamma, delta, eta, kappa and omicron variants. A list of all the considered VOCs and single-mutation variants is reported in Tables 1, 2, respectively. Starting from the X-ray structure of the WT RBD-human ACE2 complex (pdb id: 6M0J), we obtained the structure of both the 29 single-mutation variants and the seven VOCs via the computational mutagenesis protocol described in the Methods section. All complexes have been relaxed in a 100-ns-long standard molecular dynamics simulation (see Methods for simulation details). As shown in SI for all other complexes, simulations reach equilibrium after about 30 ns judging from the RMSD of the whole complex and that of the binding regions. Thus, all analyses were conducted by sampling configurations between 30 and 100 ns of simulation time.

**TABLE 1:** VOCs electrostatic properties. The mutations corresponding to the variants observed are collected together with the electrostatic character of the amino acids involved in the mutations (A: apolar, P: polar, C: charged).

Variant	Mutations	AA type	Variant	Mutations	AA Type
Alpha	N501Y	A → P		G399D	A → C
				S371L	P → A
Beta	K417N	C → A		S373P	P → A
	E484K	C → C		S375F	P → A
	N501Y	A → P		K417N	C → A
Gamma	K417T	C → P	**Omicron**	N440K	A → C
	E484K	C → C		G446S	A → P
	N501Y	A → P		S477N	P → A
Delta	L452R	A → C		T478K	P → C
	T478K	P → C		E484A	C → A
Eta	E484K	C → C		Q493R	A → C
				G496S	A → P
Kappa	L452R	A → C		Q498R	A → C
	E484Q	C → A		N501Y	A → P
				Y505H	P → C

**TABLE 2:** Binding affinity data of the considered single residue mutations. List of the experimental mutations considered in this study with their Δ*B*_*a*_ measured by Starr *et al.* (Starr et al., 2020). The mutations are divided according to the residue position they take place in.

Mutation	**Δ*B***_***a***_
K417D	−1.04
K417N	−0.45
K417T	−0.26
L452R	0.02
L455D	−2.25
L455M	0.05
F456D	−4.55
A475W	−2.26
G476P	−2.56
T478K	0.02
E484R	0.15
E484Y	−1.51
E484Q	0.03
F486D	−1.69
N487I	−2.77
C488I	−4.79
Y489S	−4.80
Q493D	−1.57
Q493M	0.18
S494D	−1.10
S494H	0.06
Y495G	−3.93
G496E	−2.33
T500I	−2.30
N501D	−2.42
N501F	0.29
N501K	−2.79
N501T	0.10
G502F	−4.80

To validate the mutational procedure, we used the experimental complexes of the SARS-CoV-2’s RBD bound to human ACE2 for gamma (PDB id: 7NXC) variant to verify that the configurations explored by the molecular dynamics simulations of the experimental complex overlap with those sampled during the MD of the computationally mutated ones. In practice, we compared the structures obtained for both simulations through a principal component analysis (PCA). Indeed, a set of configurations sampled from the simulation of the experimental structure was projected into the essential space, defined by the two principal components of the covariance matrix obtained from the trajectory of the computationally mutated complexes (see Supplementary Material S1). This test shows an overlap between the two sets of structures, which indeed have a high degree of similarity in terms of the backbone conformation confirming that, for this specific system, computational mutagenesis does produce good starting models.

### Comparison between global and local effects of mutations in terms of non-bonded interactions

To begin with, we focused on the non-bonded intermolecular interaction energies. In order to compactly schematize the complex architecture of the intermolecular interactions, we represented the protein complex as a bipartite graph (Miotto et al., 2018; Miotto et al., 2020; Miotto et al., 2022b), where each residue is a network node and couples of residues (i.e., nodes) not belonging to the same structure are connected by a weighted link if their minimum distance is lower than 12 Å. Weights are given by either the Coulombic and/or the Lennard-Jones interaction energies (see Methods for details). As a first analysis, for each complex, we evaluated the difference of the mean total Coulombic $\left(\Delta E_{C}^{\mathrm{tot}}\right)$ and Lennard-Jones $\left(\Delta E_{LJ}^{\mathrm{tot}}\right)$ energies between each variant complex and the WT one.

Note that energy differences are obtained as averages of the energies calculated on a set of configurations sampled from the equilibrium of the molecular dynamics simulation. Such descriptors indirectly take in consideration solvation effects, as protein-water interactions act influencing the motion of residues and thus the fluctuations of the computed interaction energies.

**FIGURE 1:**
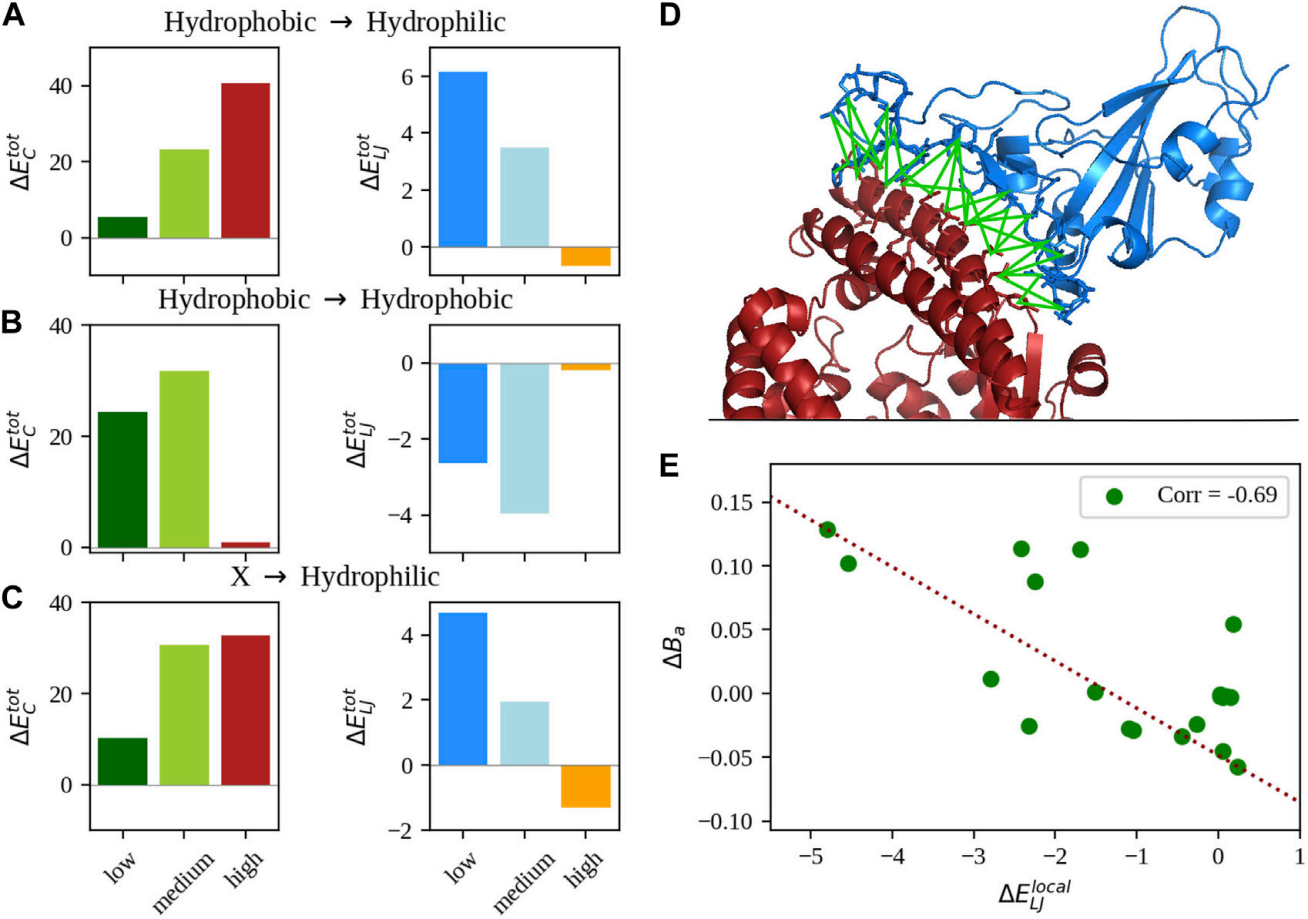
Analysis of the non-bonded intermolecular interactions. **(A)** Mean difference of total Coulombic (left) and Lennard-Jones (right) interaction energy between the single-mutation variants and the WT of the SARS-CoV-2 spike-human ACE2 complex stratified by different ranges of experimentally measured binding affinity (see Methods for details on the ranges). Only complexes whose single mutation turns a hydrophobic amino acid into a hydrophilic one were considered. **(B)** Same as in panel **(A)**, but considering only complexes where mutations preserve the hydrophobicity of the residue. **(C)** Same as in panel **(A)**, but considering complexes whose outcome of the mutation was a hydrophilic amino acid substitution. **(D)** Cartoon representation of the complex between the SARS-CoV-2 spike (in blue) and human ACE2 receptor (in red) with the interface interaction network highlighted in green. **(E)** Difference between the local mean Lennard-Jones interaction energy of the single mutation variants and the WT as a function of the difference of binding affinity. Only complexes whose mutations do not involve hydrophobic residues were considered.

More details on the calculation of these quantities are reported in the Methods. Interestingly, stratifying the dataset with respect to the hydrophobic/hydrophilic nature of the mutated amino acid, we found different trends in relation to the complexes’ binding affinities, which seem to suggest that at least two routes are possible to increase the complex stability by means of single mutations. In particular, Figure 1A shows the values of $\Delta E_{C}^{\mathrm{tot}}$ (left panel) and $\Delta E_{LJ}^{\mathrm{tot}}$ (right panel) averaged over complexes having a lower, medium lower, and higher binding affinity with respect to the WT and considering variants in which mutations turned a hydrophobic amino acid into a hydrophilic one. As one can see, the two kinds of interaction energies behave oppositely: $\Delta E_{C}^{\mathrm{tot}}$ assumes higher, positive values as the difference in binding affinity goes from much lower to higher, meaning that the total Coulombic interaction energy becomes less favorable (overall WT energy is negative). On the other hand, Lennard-Jones energy difference decreases, becoming negative for complexes with higher affinity. This means that for the affinity to increase, the complexes build more favorable Lennard-Jones interactions with respect to the WT at the cost of reducing the favorable Coulombic term. Notably, this trend is conserved whatever the starting amino acid class is (see Figure 1C). Considering mutations that preserve the hydrophobic nature of the amino acid instead, one can see (Figure 1B) that affinity only increases while the complexes maintain very similar non-bonded interaction energies. On the contrary, as such interactions vary the complexes rapidly lose stability. To further investigate the behavior of hydrophilicity-preserving mutations, we looked at the effect of the mutation on the local rearrangement of the interaction network. To do so, we evaluate for each complex the difference between the strength (see Figure 1D for a sketch and Methods for details) of the mutated residue with respect to the WT. In Figure 1E, we show the difference in binding affinity as a function of the difference in local van der Waals interaction energy (i.e., network strength) for complexes whose mutations do not involve hydrophobic amino acids. Interestingly, there is a significant anticorrelation of about −0.70 (*p*-value: 0.001), indicating that the higher the stabilization effect of the mutation, the lower the interaction energy, i.e., complexes have to be able to rearrange the binding region side chains to optimize Lennard-Jones potential energy in order to acquire a more stable complex.

### Analysis of shape and electrostatic complementarity at the binding interface

**FIGURE 2:**
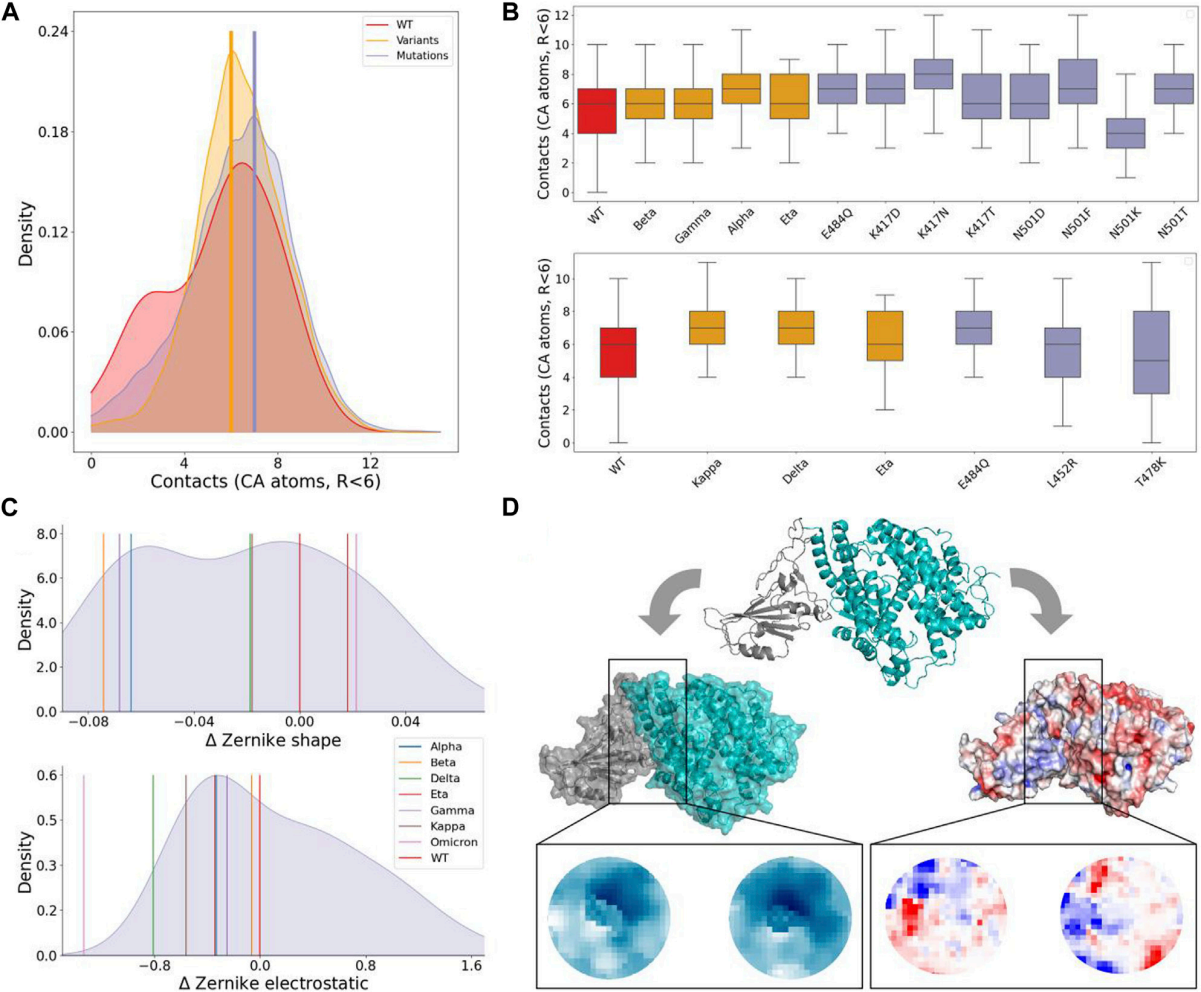
Shape and electrostatic complementarity analysis of spike-ACE2 binding region. **(A)** Density distribution of the number of contacts between spike and ACE2 residues during the simulation of the WT complex (red), the seven considered VOCs (orange), and the 29 single mutation complexes (violet). Vertical yellow and violet lines mark the modes of the VOCs and single mutation distributions, respectively. **(B)** Boxplots of the contacts between spike and ACE2. Complexes are divided according to whether mutations involve residues 484, 417, and 501 (top) whereas the plot on the bottom concerns residues 484, 452, and 478 (bottom). Boxes are colored differently when considering the WT complex (red), complexes of VOCs (orange), or single mutation complexes (violet). **(C)** Density distribution of the shape (top) and electrostatic (bottom) complementarities measured in terms of Euclidean distances between the Zernike descriptors (see Methods) for the 29 single mutation complexes reported in TableII. The Zernike distances of the wild type and seven considered VOCs are indicated by vertical colored lines. **(D)** Cartoon representation of the spike-ACE2 complex carrying mutation A475W (top) together with the corresponding molecular (left) and electrostatic (right) surfaces. Zoomed regions show the 2D projections of the interacting patches considered for the Zernike description. The shape projection is shown in a blue scale, and the colors in these planes are determined by the distance of the surface points from a predefined origin, while the electrostatic projection is shown in the blue-red scale representing the electrostatic potential values of the projected points ranging from negative to positive values of the electrostatic potential, respectively.

To further understand the changes in stability between WT, variants, and experimental mutations, we looked at the contacts (defined as the number of CA atoms on a surface closer than 6 Å to another CA atom on the second surface), which have been previously linked to binding affinity (Anna and Alexandre, 2015). Figure 2A shows the distribution of contacts for the three categories, together with the mode of the variants and mutations distributions. Figure 2B compares in detail the variants with the experimental single mutations included in them. Notably, mutation K417N has the highest number of contacts, even compared to other mutations at the same residue. It is comprised in beta, despite this variant having a lower number of contacts. Beta and gamma only differ for the substitution at position 417; even if the corresponding single experimental mutations (K417N and K417T) have different numbers of contacts, the two variants show no difference. One of the other shared mutations between beta and gamma is N501Y, which characterizes alpha as well. In this case, the substitution of asparagine with tyrosine does indeed result in the highest number of contacts, compared to other mutations at position 501. The other shared mutation between them is E484K (also appearing in the eta variant); comparing the contacts of eta with those of E484Q, it can be seen that this last substitution produces a higher number of contacts. Nevertheless, it was chosen by neither of the three variants. The kappa variant again presents the mutation E484K. In this case, it instaurates a number of contacts as higher as the one of mutation E484Q. This could depend on the presence in kappa of another mutation, L452R, that even though taken alone produces fewer contacts, when combined with E484K increases them. L452R appears in the delta as well. Even in this case, its combination with a mutation that alone would form a lower number of contacts (T478K) increases them.

Next, to compactly describe the organization of the residue side chains in the binding region, we moved to consider the molecular and the electrostatic potential surface regions of the two interacting molecules (see Methods for more details) and measured the shape and electrostatic complementarity at the interface. To do so, we selected the portions of the molecular surfaces formed by the interacting residues in the WT complex (as discussed before) and defined *N* pairs of interacting patches, where *N* corresponds to the 5% of the points forming the surface mesh included in that interacting region. Each patch is then associated with two Zernike vectors, characterizing its molecular and electrostatic potential surfaces, respectively (see (Milanetti et al., 2021b), (Grassmann et al., 2022) and Methods for more details). For each frame *i*, the distances between the Zernike shape (electrostatic) vectors of paired patches were computed resulting in the values $Z_{s}^{i}$ $\left(Z_{el}^{i}\right)$ . The smaller $Z_{s}^{i}$ , the higher the shape complementarity between the patches, whereas $Z_{el}^{i}$ similarly reflects the electrostatic complementarity. At the end of this process, Zernike shape and electrostatic distances were calculated for each complex. To evaluate the effects of mutations on the spike-ACE2 interaction, compared to WT, we computed for each of the 36 considered complexes the difference (Δ*Z*_*s*_ and Δ*Z*_*el*_) between the Zernike distances (*Z*_*s*_ and the *Z*_*el*_) of each complex and those of the WT, so that the lower the value the higher the increase in complementarity compared to WT.

Figure 2C shows the density distribution of Δ*Z*_*s*_ and Δ*Z*_*el*_ for the single mutation complexes (obtained starting from the process schematized in Figure 2D). Values relative to the considered VOCs are shown as vertical bars. Considering the experimentally measured single mutation complexes (reported in Table 2), it can be observed that they decrease the stability of the complex, with the exceptions of mutations E484R (Δ*B*_*a*_ =0.15), L455M (Δ*B*_*a*_ =0.05), Q493M (Δ*B*_*a*_ =0.18), S494H (Δ*B*_*a*_ =0.06), N501F (Δ*B*_*a*_ =0.29), N501T (Δ*B*_*a*_ =0.10), L452R (Δ*B*_*a*_ =0.02), T478K (Δ*B*_*a*_ =0.02) and E484Q (Δ*B*_*a*_ =0.03). As expected, all these mutations increase the shape complementarity, having $Z_{s}^{d}<0$ .

Interestingly, five out of the seven considered VOCs increased both their shape and electrostatic complementarities with respect to the WT. The two exceptions are the eta ( $Z_{s}^{d}=$ 0.018) and omicron ( $Z_{s}^{d}=$ 0.021) variants that only improved their electrostatic match. We note that in both cases, mutations on the binding region favored the appearance of positive charged amino acids (see Table 1): the E to K mutation of the eta variant and five out of six of the mutations in the omicron which resulted in the appearance of a charged residue.

Finally, we note that the variants with the highest shape complementarity, beta, and gamma, have the lowest electrostatic complementarity. On the other hand, it decreases for the ∼48% of the experimental variants.

**FIGURE 3:**
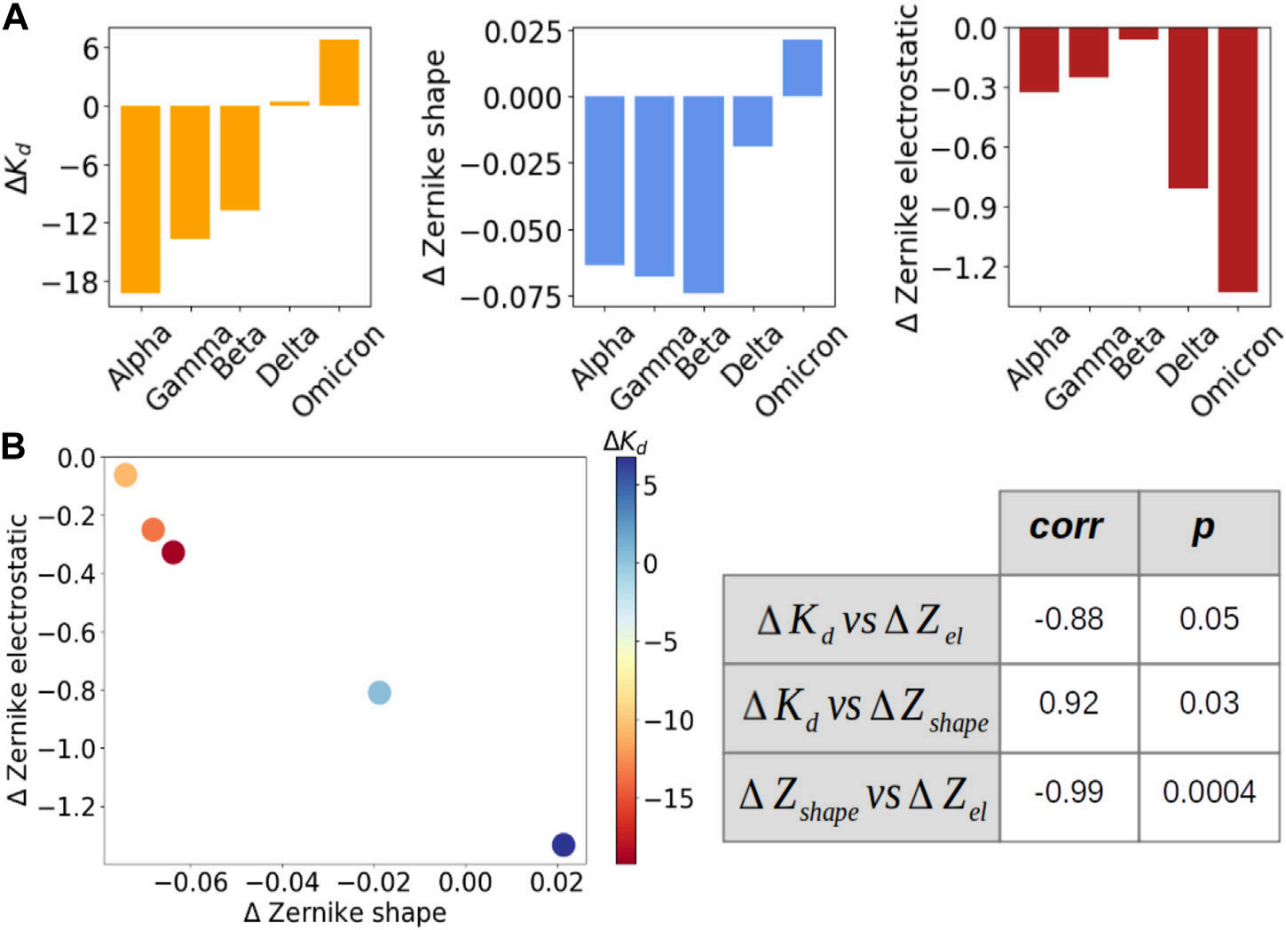
Evaluation of the correlation between dissociation constant and shape and electrostatic complementarity for five VOCs. **(A)** From left to right: Δ*K*_*d*_, Δ*Z*_*s*_ and Δ*Z*_*el*_ for five VOCs (alpha, gamma, beta, delta, omicron). The variants are ordered according to their dissociation constant value. **(B)** On the left, Δ*Z*_*el*_ as a function of Δ*Z*_*s*_ for each of the five VOCs. Each point is colored according to the Δ*K*_*d*_ value of that variant, as indicated by the color bar. On the right, the correlation (first column) and Pearson (second value) value between the three quantities are presented in **(A)**.

To better study the correlation between shape and electrostatic complementarity and complex stability, we extended our analysis on five VOCs (alpha, gamma, beta, delta, and omicron) for which the dissociation constant *K*_*d*_ has been experimentally measured (Han et al., 2022). As already done for shape and electrostatic complementarity, we computed for each variant the difference between its dissociation constant and that of WT (Δ*K*_*d*_). These values are shown in Figure 3A, together with the Δ*Z*_*s*_ and Δ*Z*_*el*_ of each variant; Figure 3B instead shows that Δ*K*_*d*_ strongly correlates with Δ*Z*_*s*_ (correlation of 0.92): as expected, more stable complexes show the highest shape complementarity at the interfaces. Interestingly, Δ*K*_*d*_ also shows a strong anti-correlation with Δ*Z*_*el*_ (correlation of −0.88): it seems that less stable complex electrostatic complementarity tends to compensate for shape complementarity. This is confirmed by the anti-correlation between Δ*Z*_*el*_ and Δ*Z*_*s*_, reaching a value of −0.99 (*p*-value at 0.0004).

### Analysis of the fluctuations of secondary structures

**FIGURE 4:**
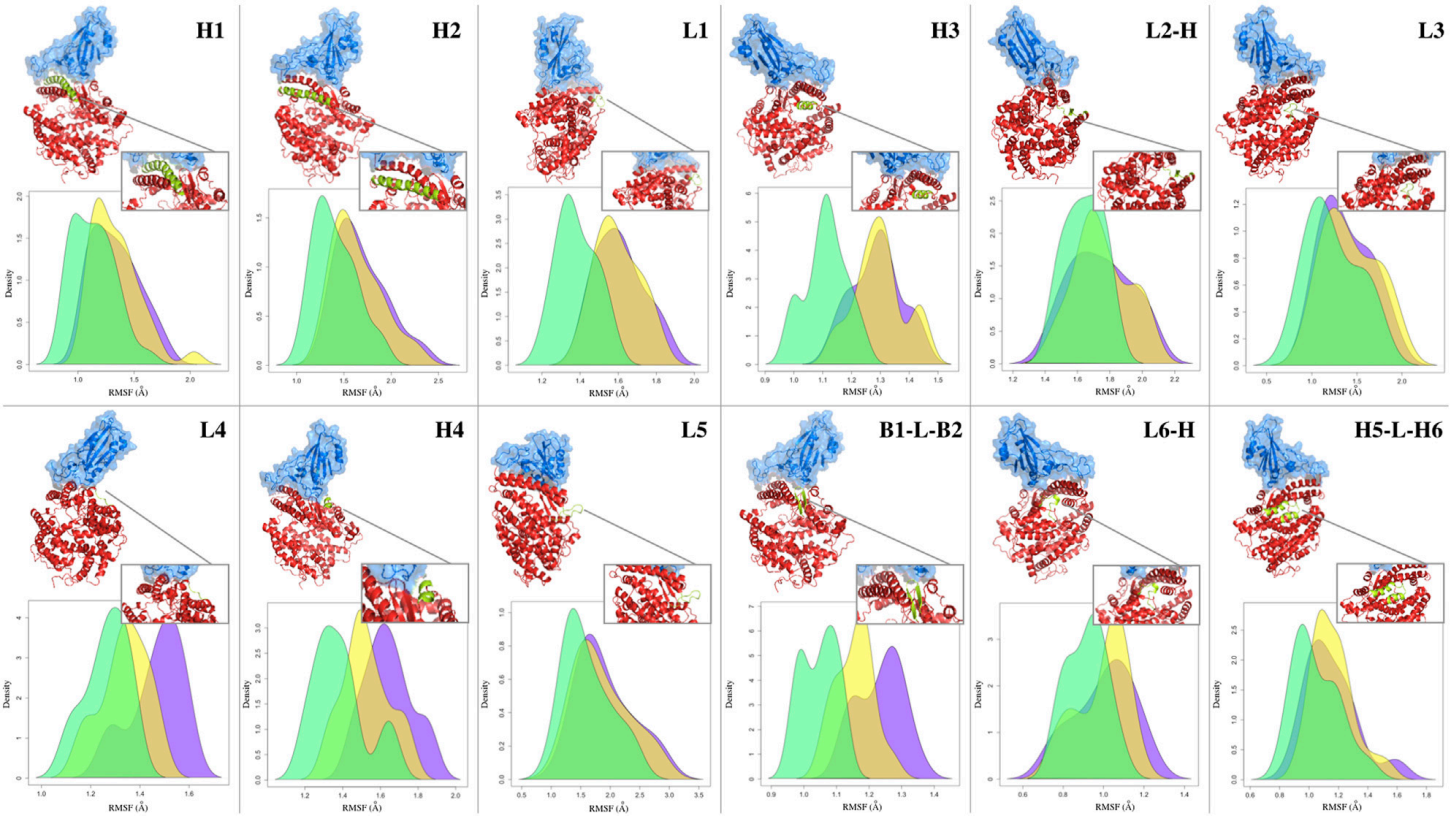
Fluctuations of ACE2 secondary structure residues. Density distribution of the RMSF of the residues forming the secondary structures of the human ACE2 receptor in complex with SARS-CoV-2 spike RBD. Cartoon representation of the complex with a zoom on the considered secondary structure is reported above the distribution panel for each considered secondary structure. Green, yellow, and purple shaded curves are given considering all residues of the single-mutation variants (see Table 2) whose difference in binding affinity (*B*_*a*_) with respect to the wild type complex is lower than −3, between −3 and −0.05, and higher than −0.05, respectively.

Typically, after the formation of the molecular complex, the binding sites of proteins decrease their degree of mobility (Teague, 2003). This introduces an entropic term in the complex binding affinity. In fact, when two proteins bind, both structures undergo a certain degree of conformational changes and become more ordered or restricted in their motions. This reduction in entropy can have an impact on the binding affinity as favorable binding interaction should not only result in a strong binding affinity but also maintain a balance between the enthalpic (energy-related) and entropic (entropy-related) contributions. To probe this aspect, we looked at a minimal descriptor explicitely accounting for local motions, i.e., the Root Mean Square Fluctuation (RMSF) of each protein residue (i.e., looking at the average mobility of the residue over the simulation time). Although the stabilizing role of the molecular partner is known, the relationship between the type of binding and the fluctuation of any other sub-region of the protein (also considering the regions not directly involved in the molecular binding) is not so trivial (Xing et al., 2016). In order to investigate the dynamic behavior of the ACE2 receptor in interaction with the different mutated forms of the spike protein, we analyzed the mean fluctuations of specific ACE2 regions. In particular, we have defined 12 sub-regions of the ACE2 protein (see Table 3), localized close to the interface but not entirely involved in the binding with the spike protein of SARS-CoV-2. Each of these regions is composed of one or more secondary structures, mostly involving loops and alpha helices. Only in one case, for the region called B1-L-B2, do we consider two beta strands, B1 and B2, joined by a short loop (L). Similarly, we have also defined a set of sub-regions for the spike protein, described in Supplementary Figure S6. The basic idea, in this analysis as well, is to investigate the relationship between the dynamic-structural properties of the interacting proteins and the binding properties, which have been described in terms of experimental binding affinity for the selected dataset of single mutations. More specifically, in order to work with average properties, we divided the dataset into three groups based on the binding affinity of each ACE2-spike system. In particular, we defined a group of complexes whose mutation produced a drastic decrease of binding affinity (‘low’, Δ*B*_*a*_ < − 3), one that resulted in a medium decrease (‘medium’, −3 < *B*_*a*_ < − 0.5) and one where mutation produced no effect or an increase of binding affinity (‘high’, Δ*B*_*a*_ > − 0.05). Therefore, we calculate the RMSF of the residues belonging to each sub-region, considering the three groups separately. In Figure 4, the three RMSF distributions for each sub-region are depicted, where we show in green, yellow, and violet the RMSF values of the residues belonging to the low, medium, and high-affinity groups, respectively. More in detail, taking advantage of the fact that the positions of the residues are conserved among the various systems, for each residue we calculate the average of the RMSF values for that specific position. Interestingly, for the ACE2-spike molecular system and for all the considered subregions, ‘low’ binding affinity systems have a lower mean fluctuation than both ‘medium’ and ‘high’ binding affinity groups. In particular, three of the identified regions, i.e., regions L4, H4, and B1-L-B2 (which involve a loop, an alpha helix, and a beta strand) present more distant RMSF distributions. To evaluate the statistical significance of the observed differences, we performed a Wilcoxon test between the distribution pairs. Specifically, combining together the three low-affinity distributions (in green), the three medium-affinity distributions (in yellow), and the three high-affinity distributions (in violet), as shown in Figure 4, we perform both (i) the Wilcoxon test between the low and medium affinity curve, obtaining a *p*-value of 0.037 and (ii) the Wilcoxon test between the medium and high-affinity curve, obtaining a *p*-value of 0.034. Note that in this case, we are determining the probability that the mean RMSF of the residues belonging to sub-regions H4, L4, and B1-L-B2 of low (medium) binding affinity systems is lower than the RMSF of the same regions of medium (high) affinity. Furthermore, in order to better characterize the differences between the residues involved in the sub-regions H4, L4, and B1-L-B2, we also consider all residues belonging to these specific regions of ACE2, without calculating their averages. The results of the three distributions are shown in Supplementary Figure S5. In this case, the *p*-values of the Wilcoxon tests are 0.009 and $<10^{-4}$ , respectively.

**TABLE 3:** ACE2 secondary structure elements. Short name, residue range, and involved secondary structure elements for the twelve sub-regions in which the human ACE2 structure has been divided for the residue fluctuation analyses.

sub-region	Residues	Involved ss
H1	1–51	alpha helix
H2	56–82	alpha helix
L1	83–90	loop
H3	91–101	alpha helix
L2-H	102–110	loop and alpha helix
L3	208–218	loop
L4	319–324	loop
H4	325–330	alpha helix
L5	331–346	loop
B1-L-B2	347–359	beta strand and loop
L6-H	386–398	loop and alpha helix
H5-L-H6	458–574	alpha helix and loop

Interestingly, the same relationship between secondary structure fluctuation and binding affinity was not found for the analyzed sub-regions of the spike protein. Indeed, as shown in Supplementary Figure S6, there are no statistically significant differences between the three groups of high, medium, and low binding affinity.

Comparing the mean RMSF of each residue composing the green and purple distributions for the three found subregions, we found that differently from other subregions, all residues present higher fluctuations in the high affinity subset (see SI). This not only means that, on average, high-affinity molecules exhibit greater movement, but also that there is an altered fluctuation effect on the entire binding motif. This could have an impact on both the correlated motions of intramolecular contacts and the protein-solvent interactions, thereby altering the dynamics and structure within the network of hydrogen bonds involving the water molecules in the first hydration shell. Thus, we speculate that such small structural motifs exhibit a synergistic higher motion, which is surely worthy of further more detailed investigations.

In addition, we note that the role of the observed fluctuations (which make the binding site less rigid), suitably placed in the three-dimensional structure of the binding site, may help maintain the van der Waals interactions with the molecular partner interface during the complex motion. In this respect, high fluctuations can result in high-affinity values in the presence of correlated motions which allow the complex to maintain stable favorable van der Waals interactions during the proteins’ motions.

**FIGURE 5:**
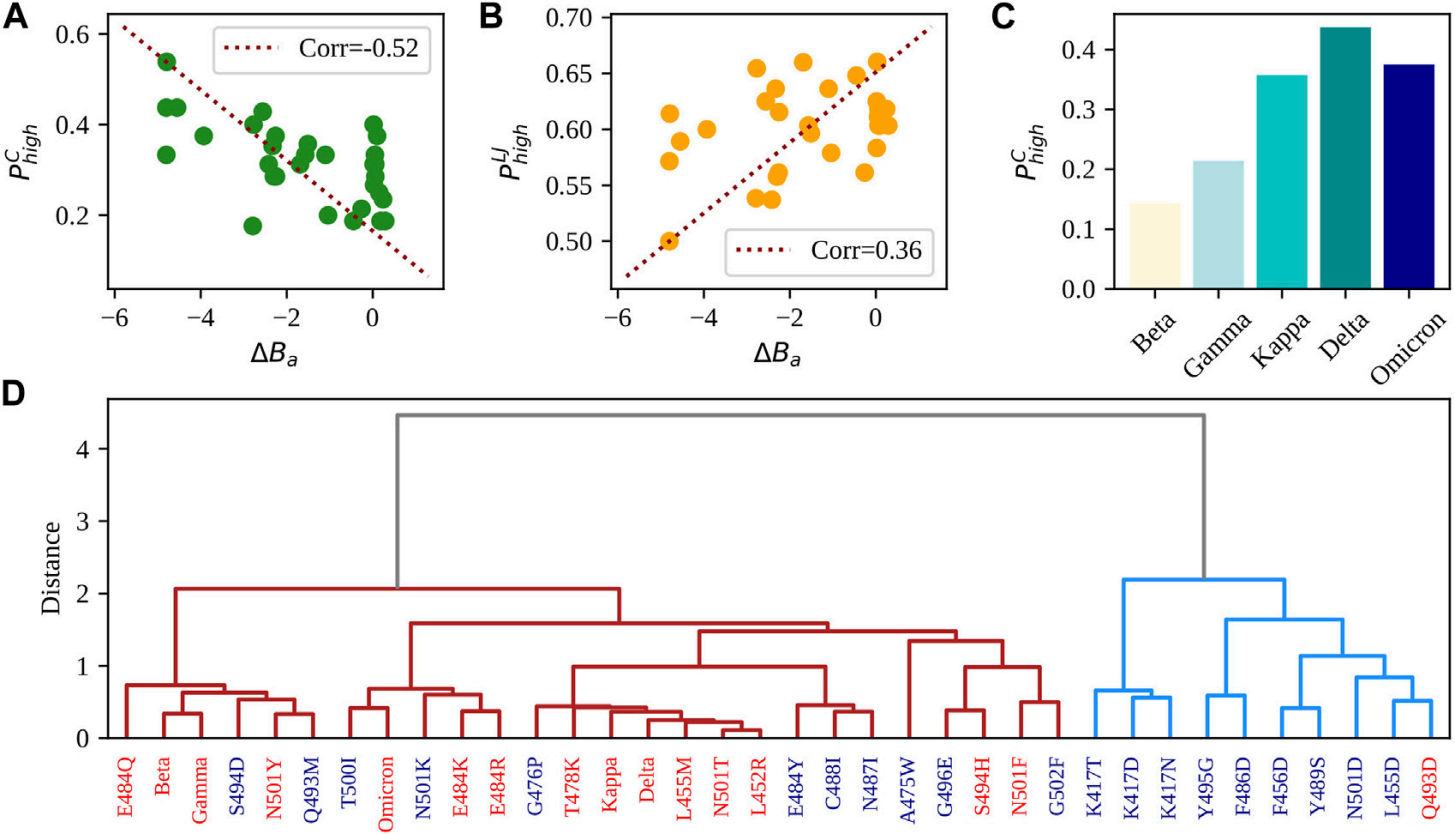
Comparison between single and multiple mutations. **(A)** Probability of finding strong Coulombic intermolecular interactions between SARS-CoV-2 RBD and human ACE2 receptor (see Methods for details on the calculation) as a function of the difference in experimental binding affinity between the single-mutation variants reported in Table 2 and the complex WT. The red dashed line represents the linear correlation axis. **(B)** Same as in panel **(A)** but considering strong intermolecular Lennard-Jones interactions. **(C)** Probability of finding strong Coulombic intermolecular interactions between SARS-CoV-2 RBD and human ACE2 receptor (see Methods for details on the calculation) for the five considered VOCs having more than one mutation on the RBD. **(D)** Hierarchical clustering of the 29 studied single-mutation variants and the seven VOCs. Variants are colored in red if their binding affinity difference is higher than −0.10. Such difference is associated with null or improved affinity with respect to the WT. When instead the binding affinity difference is lower than −0.10 (i.e., associated with a worse affinity compared to the WT), the variants are colored in blue. See Methods for details on the used clustering algorithm and descriptors.

To probe the effect of the overall higher fluctuations found in ACE2 residues, we looked at the weights of the links connecting ACE2 residues to the spike ones. First, we calculated for all the frames of the equilibrium dynamics the distance between the CA atoms of the two interacting regions and defined a contact if such distance is lower than 9 $\overset{{}^{\circ}}{A}$ ; and defined the contact probability as the number of frames in which a certain couple of residues is found in contact over the total number of frames. In SI, we reported the difference between the contact probability of each couple of residue forming the binding region of each variant and the WT one. Negative (resp. positive) values mean that the couple of residues has a contact probability lower (resp. higher) than the WT. To reduce the dimensionality of the information, we performed a principal component analysis of the considered variants. Interestingly, a difference in the PC1 component is found between complexes whose mutation produces a lowering of the stability with respect to the WT and those that present a higher affinity than the WT one (see boxplot in SI). To refine the analysis, we restricted to evaluate the probability of finding residues on the ACE2 binding region that remained strongly connected to the RBD residues during the dynamics. To do so, we defined for each residue the fraction of simulation time in which such residue shares more than three strong energetic interactions with the partner residues (see Methods for more details). In Figures 5A, B, the probabilities considering both Coulombic and Lennard-Jones interactions are plotted as a function of the complexes’ Δ*B*_*a*_. As one can see, both descriptors show a correlation with the experimental binding affinity, but while the higher the probability of finding residues strongly connected via Lennard Jones interactions the higher the resulting binding affinity, the opposite is registered for Coulombic interactions. Indeed, the Pearson correlations associated with the two interaction energies are −0.52 (*p*-value of 0.0027) and 0.36 (*p*-value of 0.047), which are meaningful for the considered number of complexes.

Moreover, in Figure 5C, we displayed the Coulombic probabilities for the studied VOCs.

### Prediction of the effects of mutations on the complex affinity

Finally, we performed a hierarchical clustering analysis combining all previously analyzed descriptors to evaluate their capability of capturing the effects of the different mutations on the complex stability. Figure 5D displays the outcome of the clustering procedure (see Methods for details on its computation). It can be seen that variants tend to divide into two groups. Interestingly, if one looks at the Δ*B*_*a*_ values of the considered 29 single-mutation variants, it turns out that the cluster on the right in Figure 5C is mostly (90%) composed of complexes with Δ*B*_*a*_ < − 0.1, while the group on the left contains all but one of the variants that increase the binding affinity and all the VOCs. Indeed, the right cluster, containing 10 proteins, includes almost exclusively complexes with lower-than-WT affinity (9), while the left cluster contains 15 complexes with higher-than-WT affinity over the total 26 protein complexes. The overall accuracy in discriminating lower/higher affinity complexes of the method is 67%. To test whether the used dynamical descriptors effectively provided more information than what one would obtain only considering standard sequence-based characteristics, we performed a clustering analysis using the charges of the interacting residues and their hydropathy index (defined by the scale provided by Di Rienzo (Di Rienzo et al., 2021)). The resulting clustering (see SI) only separates the single mutation variants from those carrying more mutations (which can have more than one different amino acid and thus a higher distance from the rest). This shows that assessing the effect of single/multiple mutations on the stability of the complex requires more complex information than those provided by standard sequence-based descriptors. In this framework, the added value of using dynamical descriptors is that they consider the interaction between the mutated residue(s) and the rest of the complex.

## Conclusion

The initial infections and the vaccination campaign have originated an immunity against the original version of the S protein. This protection can be endangered if the emerged variants are characterized by many mutations on the S protein, especially if these mutations sensibly alter the physical-chemical properties of antibody-targeted S regions (Harvey et al., 2021; Plante et al., 2021). Understanding the molecular mechanisms responsible for the fitness of novel variants is of pivotal importance, especially at the moment since new variants are rapidly emerging due to the circulation of the virus. In particular, mutations may result in very different outcomes, depending on the region of the spike where the mutation takes place and the presence of other concomitant mutations. Indeed, we know that the N501Y and E484K mutations favor the formation of a stable RBD-hACE2 complex, thus enhancing the binding affinity of RBD to hACE2. On the other hand, the K417 T/N mutation disfavors complex formation between RBD and hACE2, which has been demonstrated to reduce the binding affinity (Junker et al., 2022). However, when combined they result in an improvement in the fitness for both the beta and gamma variants. In this work, we explore from a structural point of view the rearrangements of the amino acid side chains of the RBD-ACE2 complex in a large set of single-mutation variants and in seven VOCs.

Overall, our results showed an anti-correlation between the Coulombic and Lennard-Jones energetic terms with respect to the gain in stability: mutations that increase the stability require an increase in shape complementarity and a decrease in electrostatic complementarity. Thus, an interplay between Coulombic and Lennard-Jones interactions must take place for the variant to achieve a higher affinity with respect to the WT. Different analyses have been conducted, both on the organization of the inter- and intramolecular interactions between the ACE2 receptor and the spike protein of SARS-CoV-2. Measuring the differences between the local mean Lennard-Jones interaction energy of the single mutation variants and the WT, the shape and electrostatic contribution at the interface which are calculated with Zernike formalism-based approach, and the fluctuation analysis of ACE2 receptor secondary structures from molecular dynamics simulation data, we find differences between the ACE2-spike systems each characterized by a specific experimental binding affinity, that combined together allow to estimate the outcome of point mutations on the complex binding region with a performance of 0.7. Ultimately, the relationship between ACE2-spike binding affinity and the key properties identified in this work may serve to estimate the stability of novel variants of interest as much as be used to better understand the binding mechanisms of protein-protein complexes under mutations.

## Materials and methods

### Datasets

The collected dataset consisted of a number of mutant variants obtained from the experimentally resolved structure of the WT spike protein bound to ACE2 (PDB id: 6M0J). Indeed, all the variants structures were derived by subjecting the WT to computational mutagenesis performed via the dedicated tool provided in the PyMol software (Schrödinger and Warren, 2000). Specifically, the dataset accounted for 31 mutations with known experimental binding affinity, which were provided by Starr *et al.* (Starr et al., 2020) and by Miotto *et al.* (Miotto et al., 2022a). The seven mutations related to the VOCs observed during the pandemic were also considered.

### Non-bonded energy calculation

The partial charges were assigned to atoms using the PDB2PQR software (ToddDolinsky et al., 2007), with standard options. Before the proper energy calculation, the structures were minimized with Gromacs 2020.6 (David Van et al., 2005).

The intermolecular interactions were computed employing the parameters provided by the CHARMM force field (Vanommeslaeghe et al., 2010). Specifically, given two atoms, *l* and *m*, with partial charges *q*_*l*_ and *q*_*m*_, they will interact electrostatically through the following Coulomb law:

$$E_{lm}^{C}=\frac{1}{4\pi \epsilon_{0}}\frac{q_{l}q_{m}}{r_{lm}},$$ (1)

where *r*_*lm*_ is the distance between the two atoms, and *ϵ*_0_ is the vacuum permittivity.

The Lennard-Jones potential is defined as follows:

$$E_{lm}^{LJ}=\sqrt{\epsilon_{l}\epsilon_{m}}\left[{\left(\frac{R_{\min}^{l}+R_{\min}^{m}}{r_{lm}}\right)}^{12}-2{\left(\frac{R_{\min}^{l}+R_{\min}^{m}}{r_{lm}}\right)}^{6}\right],$$ (2)

where *ϵ*_*l*_ and *ϵ*_*m*_ are the potential well depths for *l* and *m*, respectively. $R_{\min}^{l}$ and $R_{\min}^{m}$ represent the distances of the potential minima.

Summing over all the atoms pairs, the total interaction energy between residue *i* and residue *j* can be worked out as:

$$E_{AA_{ij}}^{X}=\overset{N_{\mathrm{atom}}^{i}}{\underset{l=1}{\sum }}\overset{N_{\mathrm{atom}}^{j}}{\underset{m=1}{\sum }}E_{lm}^{X},$$ (3)

where *X* indicates either the Coulombic (*X* = *C*) or Lennard-Jones (*X* = *LJ*) interaction.

### Strength

Thinking of residues in a protein as nodes in a network and of energies as the weights of the links connecting couples of nodes, the node strength can be defined as follows (Miotto et al., 2018):

$$s_{i}=\overset{N_{aa}^{i}}{\underset{j=1}{\sum }}E_{ij}$$ (4)

### Computation of network descriptors

For each ACE2-spike molecular system, three indices were calculated, which indicate the percentage of strongly interacting residues. In particular, we focused the analysis only considering the ACE2 binding site, in order to measure how many residues of the ACE2 receptor participate in binding with the SARS-CoV-2 spike protein. The three descriptors were defined as follows.

• Descriptor based on Coulomb interactions: percentage of involved residues that have more than three strong intermolecular Coulomb interactions with spike protein residues. A Coulomb interaction has been considered strong if its residue-residue energy, considering the sum of all the atom-atom interactions belonging to the two interacting residues, is lower than −5.5 kcal/mol.
• Descriptor based on Lennard-Jones interactions: percentage of involved residues that have more than three strong intermolecular Lennard-Jones interactions with spike protein residues. A Lennard-Jones interaction has been considered strong if its residue-residue energy, considering the sum of all the atom-atom interactions belonging to the two interacting residues, is lower than −0.025 kcal/mol.
• Descriptor based on residue-residue contact probability: percentage of residues belonging to ACE2 that have more than three highly probable interactions (during simulation) with spike protein residues. In this case, we consider two residuals with a high probability of interacting if the probability of contact is higher than 0.5.

### Molecular dynamics simulations

To perform simulations of the spike trimers Gromacs 2020.6 was used (Van Der Spoel et al., 2005), with the CHARMM-36 force field (Brooks et al., 2009). Proteins were placed in a dodecahedron simulative box, with periodic boundary conditions. Water molecules were represented according to the TIP3P model. (Jorgensen et al., 1983). Terminals were capped with −*COOH* and −*NH*_2_ groups. In all the systems, all protein atoms were at least at a distance of 1.1 nm from the box borders. The minimizations were carried out using the steepest descent algorithm. Next, a two-step equilibration of the system was run in NVT and NPT environments each for 0.1 ns at 2 fs time-step. The v-rescale thermostat was adopted to keep the temperature at the constant value of 300 K. In the production runs of 100 ns, the pressure was set at 1 bar with the Parrinello-Rahman barostat (Parrinello and Rahman, 1980). We used the LINCS algorithm (Hess et al., 1997) to constrain bonds involving hydrogen atoms. We put a cut-off of $12\overset{{}^{\circ}}{A}$ to account for short-range non-bonded interactions. On the other hand, the Particle Mesh Ewald method (Cheatham et al., 1995) was adopted for long-range electrostatic interactions. The RMSD and RMSF curves obtained after the production runs clearly showed that all the systems had reached equilibrium (see SI).

### Patches definition

All the molecular surfaces in this work were computed using the DMS software with standard parameters (Richards, 1977).

The centers of the patches were defined using the starting structure of the spike protein original version, sampling one point per ${\overset{{}^{\circ}}{A}}^{2}$ from the molecular surface of such structure. Each of the resulting 27,179 points was used to build a patch. In the starting structure of the WT spike protein, a patch is defined as the set of molecular surface points closer than 6 $\overset{{}^{\circ}}{A}$ to the patch center. To determine the patch centers in all the other simulation frames and for the variants, we super-positioned each structure with the starting structure of the original spike protein. The points closest to the ones selected on this original version were taken as the patches center of that structure. The patch was then constructed using the same threshold of 6 $\overset{{}^{\circ}}{A}$ .

### Zernike descriptors

The points composing a patch can be projected with a conical symmetry onto a plane, in such a way that the geometrically relevant information is maintained (Milanetti et al., 2021b). This allows each patch to be expressed in terms of a 2D function *f*(*r*, *ϕ*) defined in the unitary circle(region *r* < 1), which can in its turn be expanded in the Zernike polynomials basis:

$$f\left(r,\phi \right)=\overset{\infty }{\underset{n=0}{\sum }}\overset{m=n}{\underset{m=0}{\sum }}c_{nm}Z_{nm},$$ (5)

where

$$\begin{array}{ll} c_{nm}= & \frac{\left(n+1\right)}{\pi }\left\langle Z_{nm}|f\right\rangle \\ & =\frac{\left(n+1\right)}{\pi }\int_{0}^{1}drr\int_{0}^{2\pi }d\phi Z_{nm}^{*}\left(r,\phi \right)f\left(r,\phi \right) \end{array}$$ (6)

are the expansion coefficients, also referred to as the Zernike moments. *Z*_*nm*_(*r*, *ϕ*) are the Zernike polynomials, consisting of a radial and an angular factor:

$$Z_{nm}=R_{nm}\left(r\right)e^{im\phi }.$$ (7)

The radius dependence, given *n* and *m*, can be obtained through the following expression:

$$R_{nm}\left(r\right)=\overset{\frac{n-m}{2}}{\underset{k=0}{\sum }}\frac{{\left(-1\right)}^{k}\left(n-k\right)!}{k!\left(\frac{n+m}{2}-k\right)!\left(\frac{n-m}{2}-k\right)!}r^{n-2k}$$ (8)

For each couple of polynomials, the following holds:

$$\left\langle Z_{nm}|Z_{n^{\prime }m^{\prime }}\right\rangle =\frac{\pi }{\left(n+1\right)}\delta_{nn^{\prime }}\delta_{mm^{\prime }}$$ (9)

This result ensures that the set of polynomials forms a basis. Therefore, knowing all the coefficients {*c*_*nm*_} it is possible to recover the original function, while the detail level of the description is determined by the order of expansion, *N* = max(n).

It can be shown that the modulus of a coefficient (*z*_*nm*_ = |*c*_*nm*_|) is invariant under rotations around the origin, thus turning out to be independent of the phase. Consequently, the *z*_*nm*_ are referred to as the Zernike invariant descriptors.

The shape similarity between two patches is therefore assessed by comparing their Zernike invariants. In particular, the similarity between two patches *i* and *j* is measured as the Euclidean distance between their invariant vectors. We adopted an expansion order N=20 which therefore led to 121 invariant descriptors for each patch.

### Clustering procedure

We clustered the descriptors, i.e., the local averaged Coulombic energy, $E_{c}^{\mathrm{tot}}$ , the local averaged Lennard Jones energy, $E_{LJ}^{\mathrm{tot}}$ , the minimum average distance *D*_*min*_, the probability of nodes to be connected *P*_*ij*_, the Zernike distance *Z*_*s*_, the probability of having ACE2 residues with high Coulombc interactions, $P_{\mathrm{high}}^{C}$ , the probability of having ACE2 residues with high Lennard Jones interactions, $P_{\mathrm{high}}^{LJ}$ , the probability of having ACE2 residues with a high number of close spike residues $P_{\mathrm{high}}^{D}$ and the mean RMSF of each complex using the Euclidean distance and the Ward method as linkage function, via the ‘linkage’ function of the ‘cluster. hierarchy’ package of Python Scipy.

## Data Availability

The original contributions presented in the study are included in the article/Supplementary Material, further inquiries can be directed to the corresponding author.
