# Supplementary material for: Differences in the organization of interface residues tunes the stability of the SARS-CoV-2 spike-ACE2 complex
Source: Front Mol Biosci. 2023 Jun 27;10:1205919. doi: 10.3389/fmolb.2023.1205919 (PMC10333926; doi:10.3389/fmolb.2023.1205919)
Supplement: Supplementary file 1 [file DataSheet2.PDF]

Supplementary Information
Differences in the organization of interface residues tunes the stability of the SARS-CoV-2 spike-ACE2 complex

Mattia Miotto*,[1] Lorenzo Di Rienzo,[1] Greta Grassmann,[1,2] Fausta Desantis,[1,3] Gianluca Cidonio,[1] Giorgio Gosti,[1,4] Marco Leonetti,[1,4] Giancarlo Ruocco,[1,5] and Edoardo Milanetti[1,5]

[1] *Center for Life Nano-& Neuro-Science, Istituto Italiano di Tecnologia, Viale Regina Elena 291, 00161 Rome, Italy*

[2] *Department of Biochemical Sciences Alessandro Rossi Fanelli, Sapienza University of Rome, Piazzale Aldo Moro 5, 00185 Rome, Italy*

[3] *The Open University Affiliated Research Centre at Istituto Italiano di Tecnologia, Via Morego, 30, Genova,I16163, Italy*

[4] *Soft and Living Matter Laboratory, Institute of Nanotechnology, Consiglio Nazionale delle Ricerche, 00185, Rome, Italy*

[5] *Department of Physics, Sapienza University of Rome, Piazzale Aldo Moro 5, 00185 Rome, Italy*

A. Analysis of the effect of mutations of SARS-CoV-2 spike on the affinity with ACE2 receptor

To begin with, we analyzed the experimental data provided by Starr *et al.* [1] in which the binding affinity between the RBD and ACE2 complex (see Figure 1a) was probed in a mutational scanning experiment with all possible single-mutation variants of the WT RBD. Data provided by Starr and coworkers consist in the average difference between the binding affinity (in log scale) of the single-mutant variant and the WT one (ΔB_a), thus positive values are associated with complexes with higher affinity with respect to the WT. Interestingly, computing the probability that a mutation at a certain position of the RBD sequence produces an increase of the affinity, one finds that such probabilities tend to be higher in regions that are in close proximity (e.g. closer than 10 Å) to the ACE2 receptor (see Figure 1b). On the other hand, mutating residues far (e.g. at a distance higher than 20 Å) from the binding region tends not to produce any effects on the complex stability. This can be seen from the plots in Figure 1b, where the probability of a mutation to produce an increase of the stability, i.e. $\Delta B_a > 0.05$, or not to influence the stability ($|\Delta B_a| < 0.05$) as a function of the spike RBD residues are shown, together with the distance that each residue has from the closest atom of the ACE2 receptor. Distances are averaged over the equilibrium frames extracted from a molecular dynamics simulation of the RBD-ACE2 WT complex (pdb id: 6M0J). Equilibrium frames were sampled from the trajectory neglecting the first 30 ns after checking the Root Mean Square Deviation (RMSD) of the complex (Figure 1c).

To quantify the observation that mutations producing an increase in complex stability are preferentially located near the binding region, we computed the Pearson correlation coefficient between the probability of producing an increase in the affinity and the distance of the mutated residue with respect to the partner molecule. The resulting correlation is 0.45 (p-value: 0.02); while the correlation between the probability of a mutation to produce little to no effect on the binding affinity and the distance, is 0.40 with a p-value lower than 0.0001.

Next, we computed the probabilities of increasing or decreasing the binding affinity with the partner, stratifying the data according to the type of mutation: we divided amino acids into three classes (polar, P, apolar, A, and charged, C) and stratified the data in the nine possible kinds of mutation that starting from a class of amino acid ends into another. Note that the biding interface include two ionizable residues, namely K417 and E484. To correctly assign the protonation state of such residues the propKa web-server has been used. Results are reported in Figure 1d. For instance, the group 'AA' represents all the single mutations that involve a starting residue having an apolar amino acid that is mutated into another apolar amino acid, and so on. Note that probabilities are normalized over each of the 9 kinds of amino acid substitutions and each distance interval. It is interesting to note that mutations involving charged residues have the highest probabilities to produce an increase in the binding affinity at close distances from the partner molecule (red bars in Figure 1d). Overall, this preliminary analysis confirms that mutations taking place in the surroundings of the RBD-ACE2 binding region are the ones having higher probability of producing viral strains with higher binding affinity. Thus, we chose to focus our subsequent analyses on residues of the binding regions (i.e. having distances lower than 10 Å from ACE2).

* Corresponding Author: mattia.miotto@roma1.infn.it

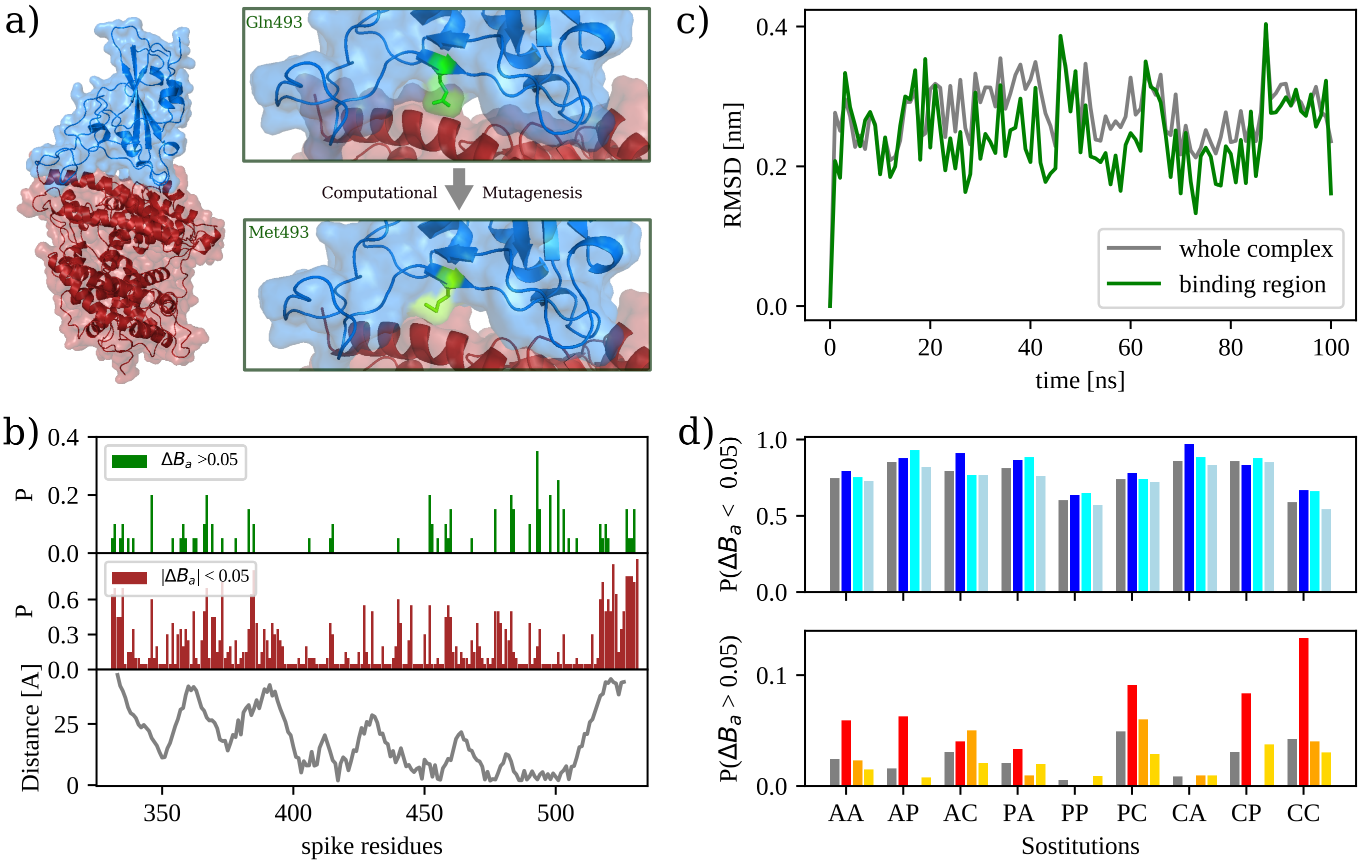

FIG. 1: **Analysis on the effect of single mutations on the SARS-CoV-2 spike protein. a)** Cartoon representation of the RBD of SARS-CoV-2 spike protein, in blue, in complex with human ACE2 receptor, in red (pdb id: 6M0J). The molecular surface of the two proteins is highlighted in shaded colors. Starting from the WT complex, single mutation variants were obtained computationally mutating the residue each time as described in the Method section. **b)** From top to bottom: probability of obtaining an increase in the binding affinity of the complex as a function of the mutated spike RBD residue, probability of obtaining a similar value of binding affinity, and mean minimum distance between each residue of the spike protein and the closest atom of the human ACE2 receptor during a molecular dynamics simulation. Probabilities were computed starting from the data published by Starr et al. [1] and range from 0 to 1, thus 0.4 means that there is 40% of probability that a mutation in a certain position will produce an increase of the complex binding affinity. **c)** RMSD as a function of the simulated time for the whole WT complex (grey) and only considering the binding region (green). **d)** Probability of obtaining a lower (top) or higher (bottom) binding affinity with a single mutation that turns an apolar (A), polar (P) or charged (C) amino acid residue into either an apolar, polar or charged one. For each combination, colored bars represent the probability stratified according to the minimum mean distance of the involved amino acid with ACE2, while grey bars represent the probability considering all possible distances. Colors range from dark blue to light blue and from dark red to yellow as the distance increases. Probabilities are normalized over each distance range and substitution class.

I. SEQUENCE-BASED DESCRIPTORS ANALYSIS

To demonstrate the importance of inspecting the dynamics of the complexes and the value of the information the simulations provide, we performed an analysis using static, sequence-based descriptors such as the charge of the residues and their hydropathic character. Specifically, we considered the residues belonging to the RBD interacting with ACE2 for the WT, the variants coming from single mutations and the VOCs displaying multiple mutations. We assessed the charge of the selected residues besides associating each of them to a hydropathy index in order to indirectly account for the behaviour of the side chains when interacting with the surrounding solvent. The hydropathy index was assigned according to the scale proposed by Di Rienzo et al.[2], in which ILE is the most hydrophobic amino acid while GLU displays the most hydrophilic character.

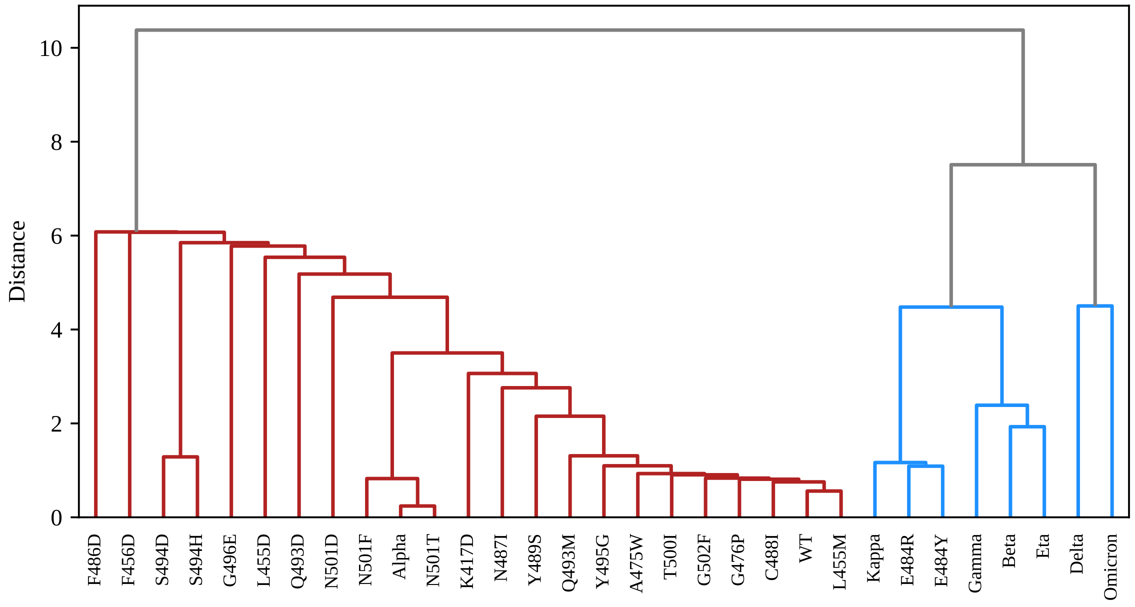

FIG. 2: Hierarchical clustering of the 29 studied single-mutation variants and the seven VOCs using interacting residue charges and hydropathy to compute the distance matrix.

Additional figures

[1] T. N. Starr, A. J. Greaney, S. K. Hilton, D. Ellis, K. H. Crawford, A. S. Dingens, M. J. Navarro, J. E. Bowen, M. A. Tortorici, A. C. Walls, et al., Cell **182**, 1295 (2020).

[2] L. Di Rienzo, M. Miotto, L. Bò, G. Ruocco, D. Raimondo, and E. Milanetti, Frontiers in molecular biosciences **8**, 626837 (2021).

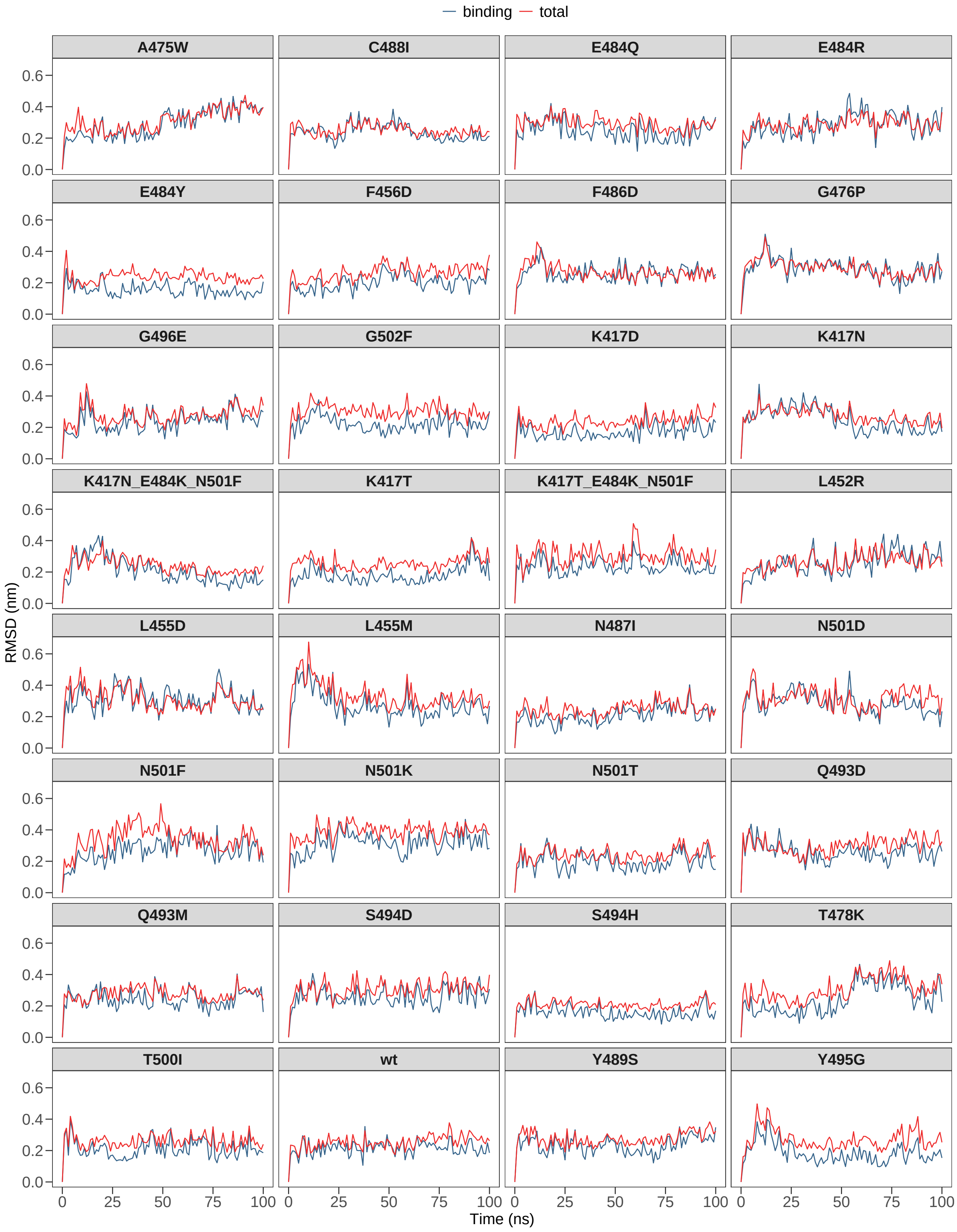

FIG. 3: **RMSD of the 31 mutants.** The RMSDs of all the variants obtained from the WT (see Methods) with respect to the simulating time are displayed. In each plot, the RMSD of the whole structure (blue) of each mutant and the RMSD of the binding site are compared (red). The set of plots also includes the RMSD of the WT.

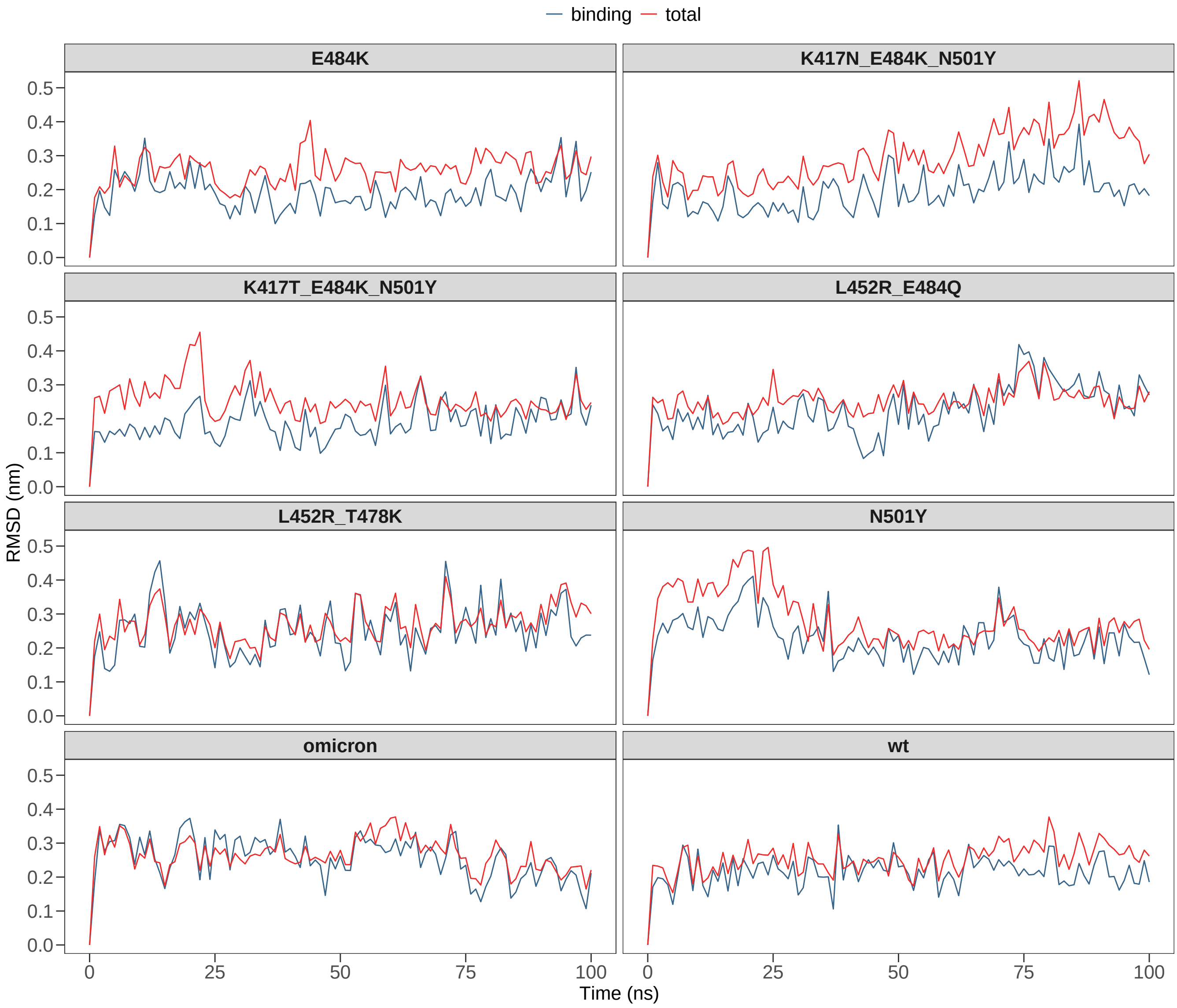

FIG. 4: **RMSD of the real variants.** The RMSDs of the variants observed during the pandemic with respect to the simulating time are displayed. In each plot, the RMSD of the whole structure (blue) of each mutant and the RMSD of the binding site (red) are compared. The set of plots also includes the RMSD of the WT.

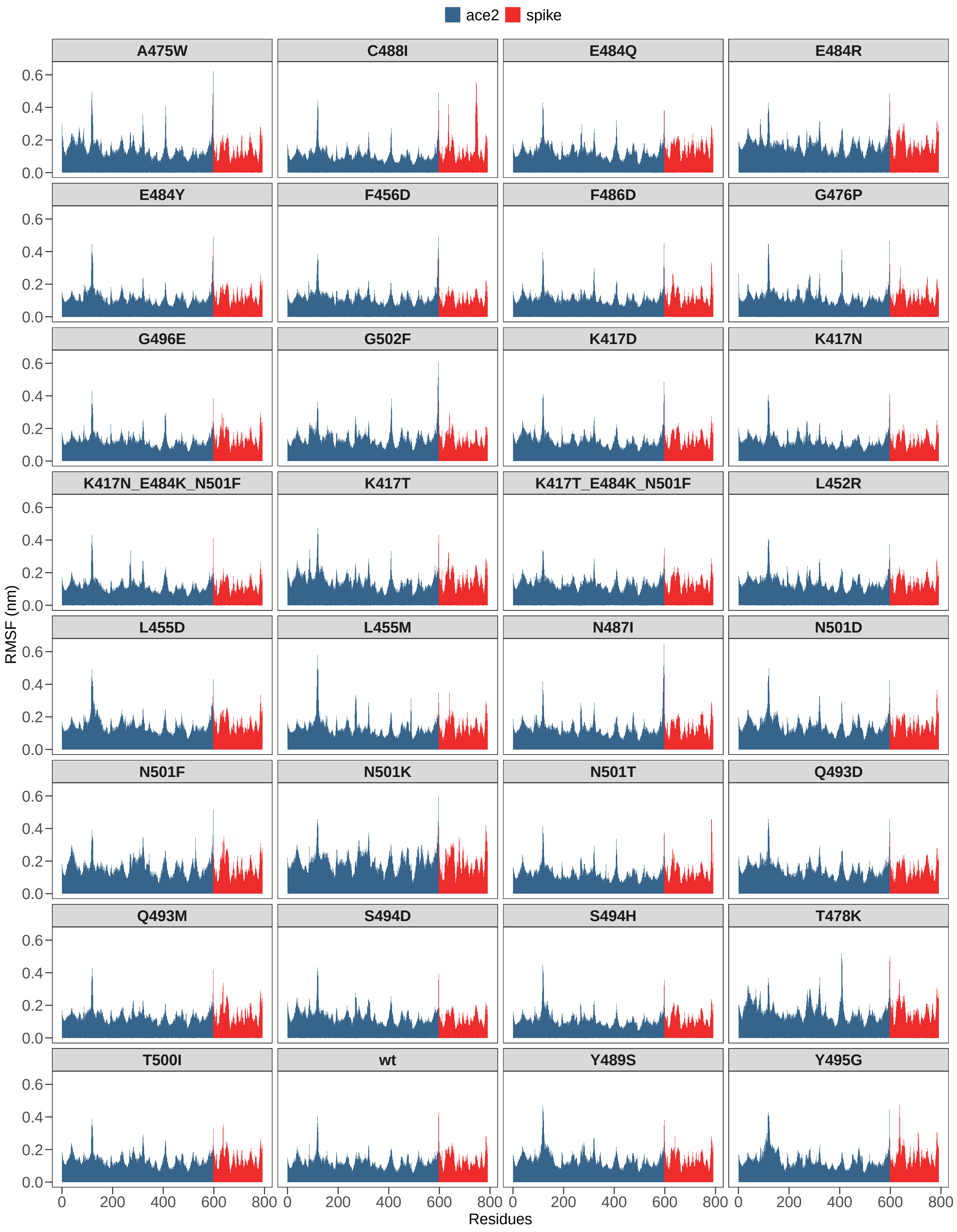

FIG. 5: **RMSF of the 31 mutants.** The RMSFs of all the variants obtained from the WT (see Methods) with respect to the simulating time are displayed. In each plot, the RMSF of ACE2 (grey) and the RMSF of the Spike protein (red) are compared. The set of plots also includes the RMSFs of the WT.

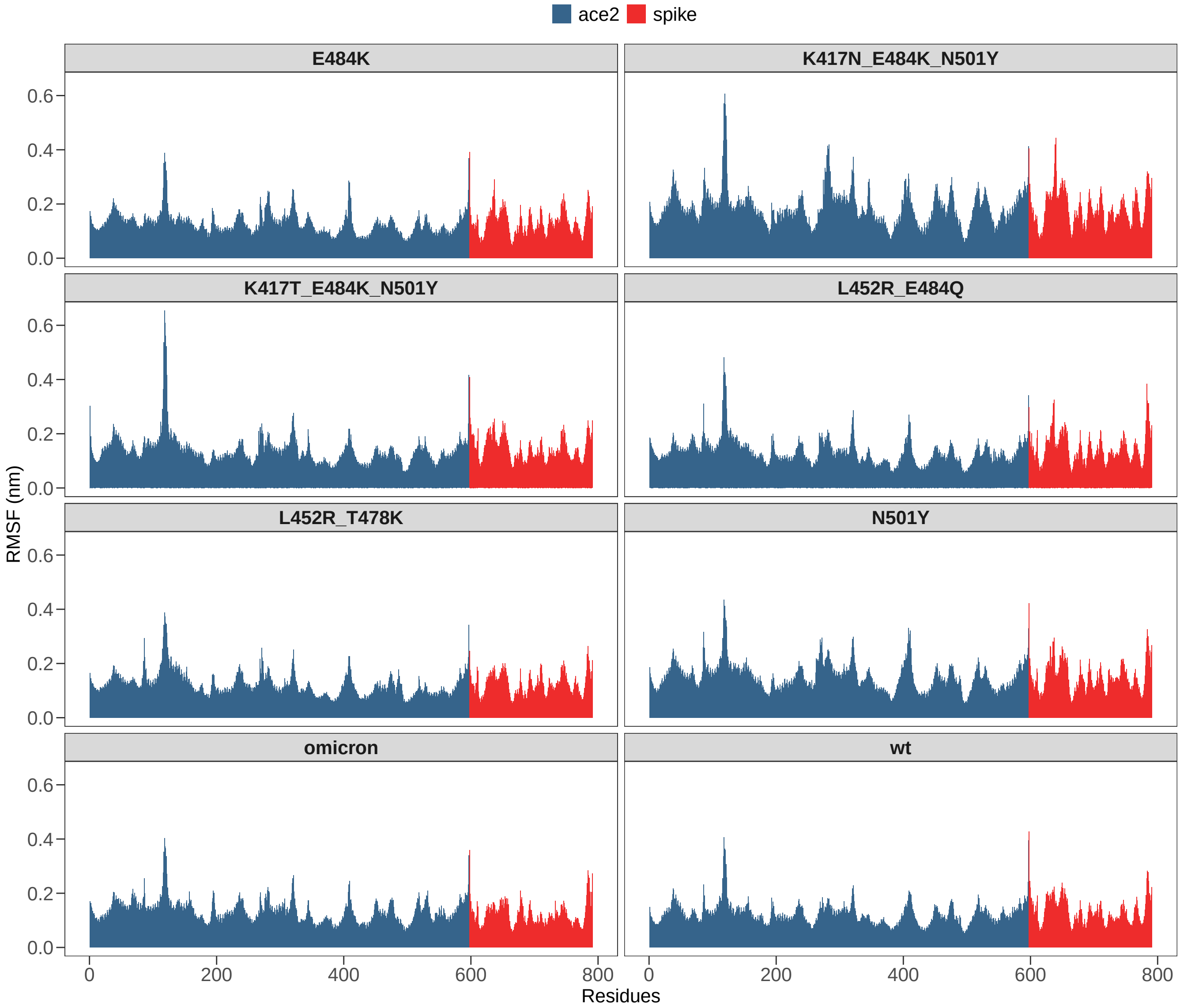

FIG. 6: **RMSF of the real variants.** The RMSFs of the variants observed during the pandemic with respect to the simulating time are displayed. In each plot, the RMSF of ACE2 (grey) and the RMSF of the Spike protein (red) are compared. The set of plots also includes the RMSFs of the WT.

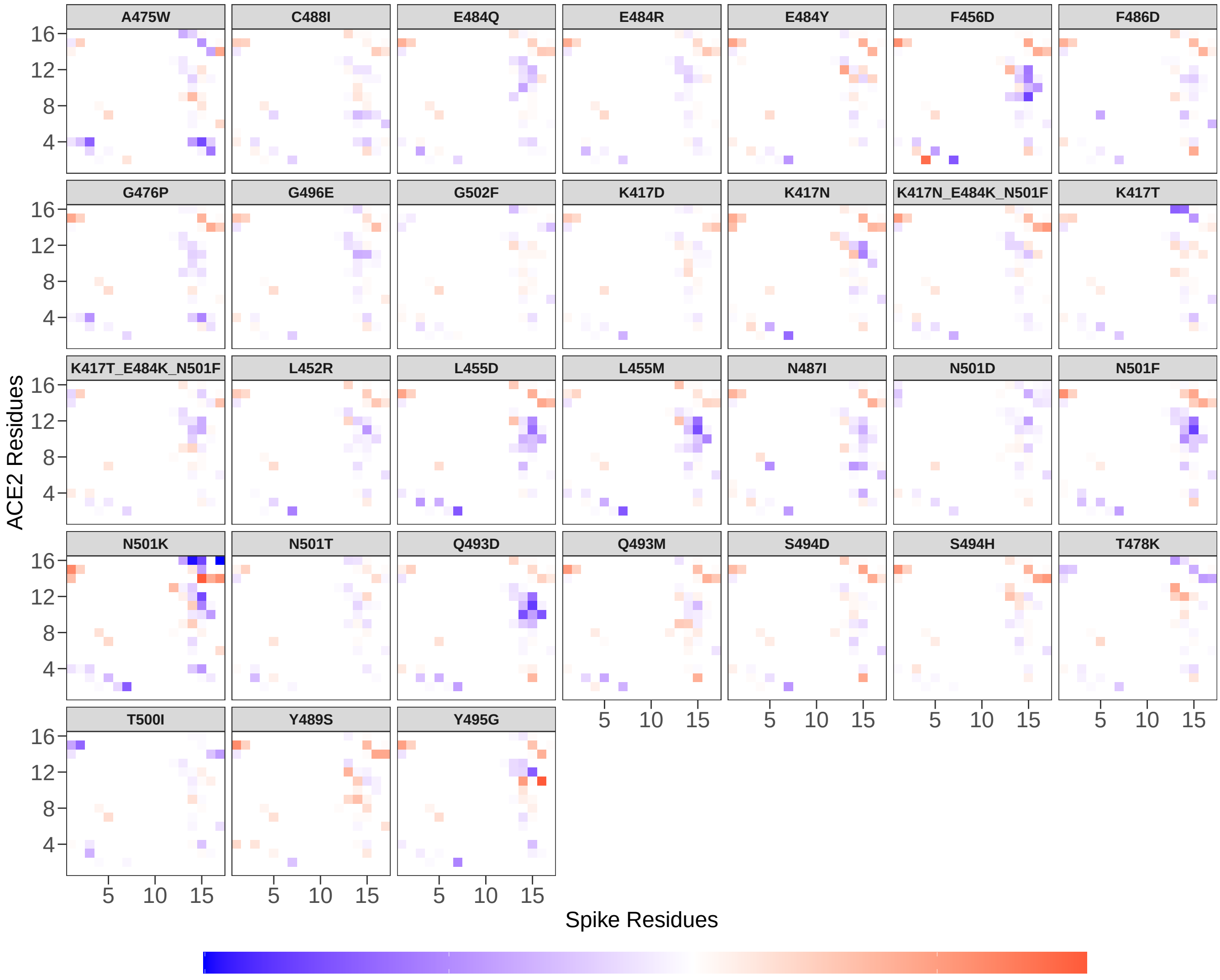

FIG. 7: **Contact probabilities for the single mutation variants.**

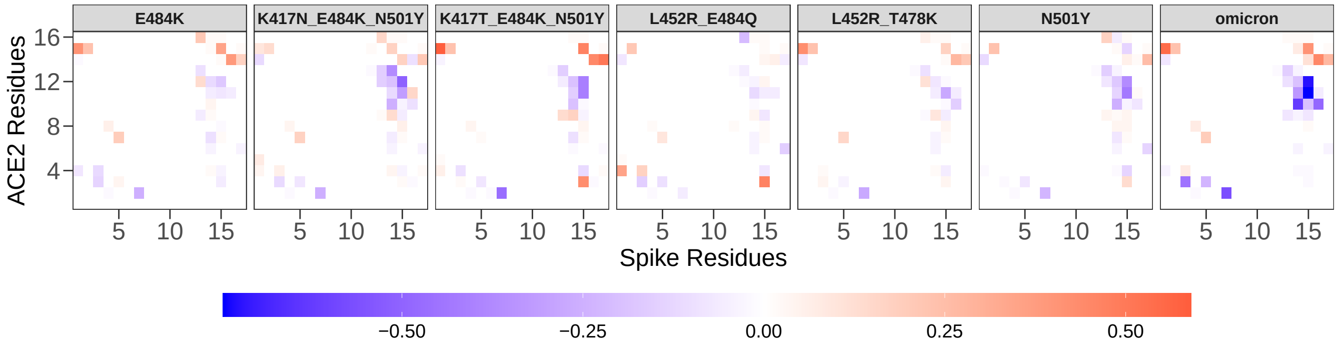

FIG. 8: **Contact probabilities for the real variants.**

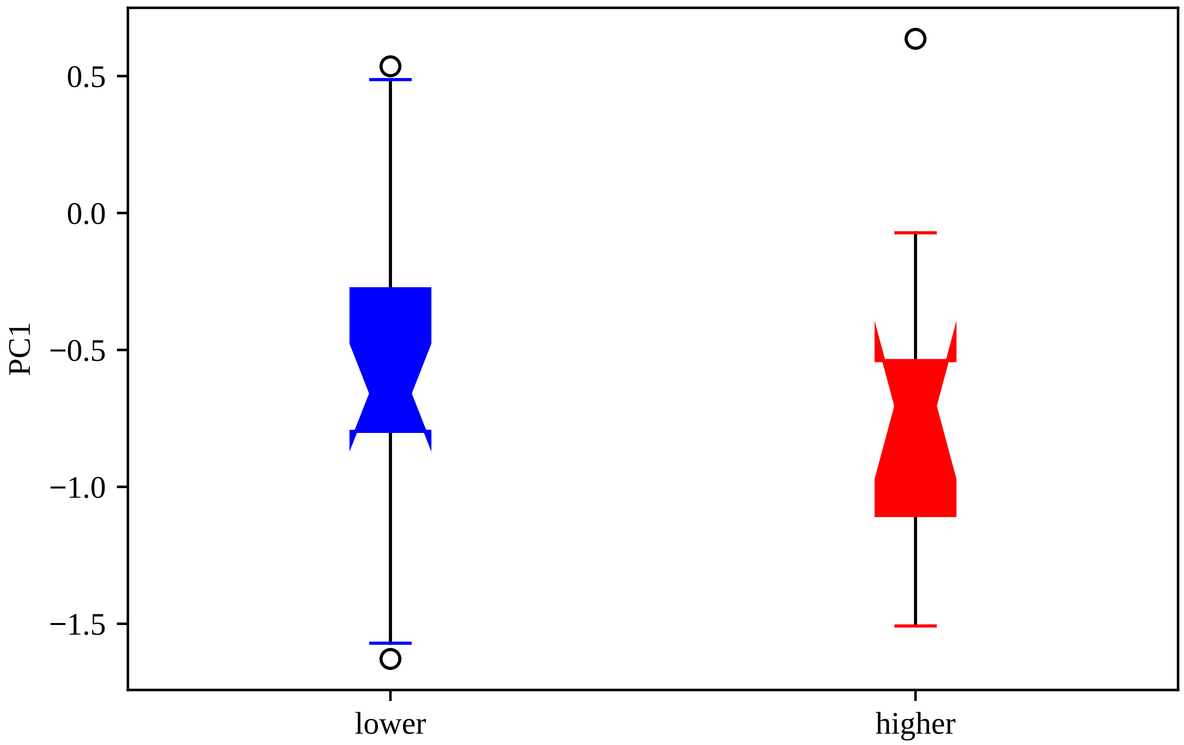

FIG. 9: **Box plot of the PC1 components of a PCA analysis over difference of the the variant contact probabilities with respect to the WT.**

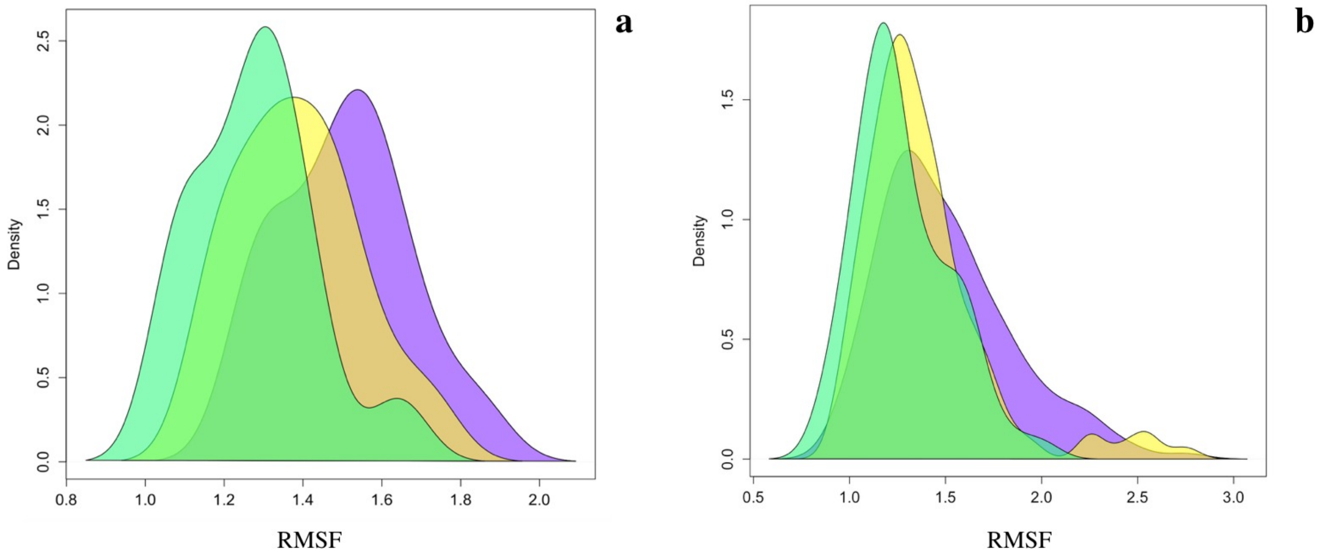

FIG. 10: **Fluctuations of ACE2 residues belonging to H4, L4 and B1-L-B2 secondary structures** Density distribution of the Root Mean Square Fluctuation (RMSF) of all residues belonging to H4, L4 and B1-L-B2 secondary structures of ACE2 receptor. Green, yellow and purple shaded curves are given considering all residues of the single-mutation variants whose binding affinity is lower than -3 kcal/mol, between -3 kcal/mol and -0.05 kcal/mol, and higher than -0.05 kcal/mol, respectively. **a** The three distributions are calculated considering all H4, L4 and B1-L-B2 residues, performing for each residue the average of RMSF with the other residues taking place in the same position and belonging to the same binding affinity group. **b** The three distributions are calculated considering all H4, L4 and B1-L-B2 residues, without calculating the average of RMSF for each residue position.

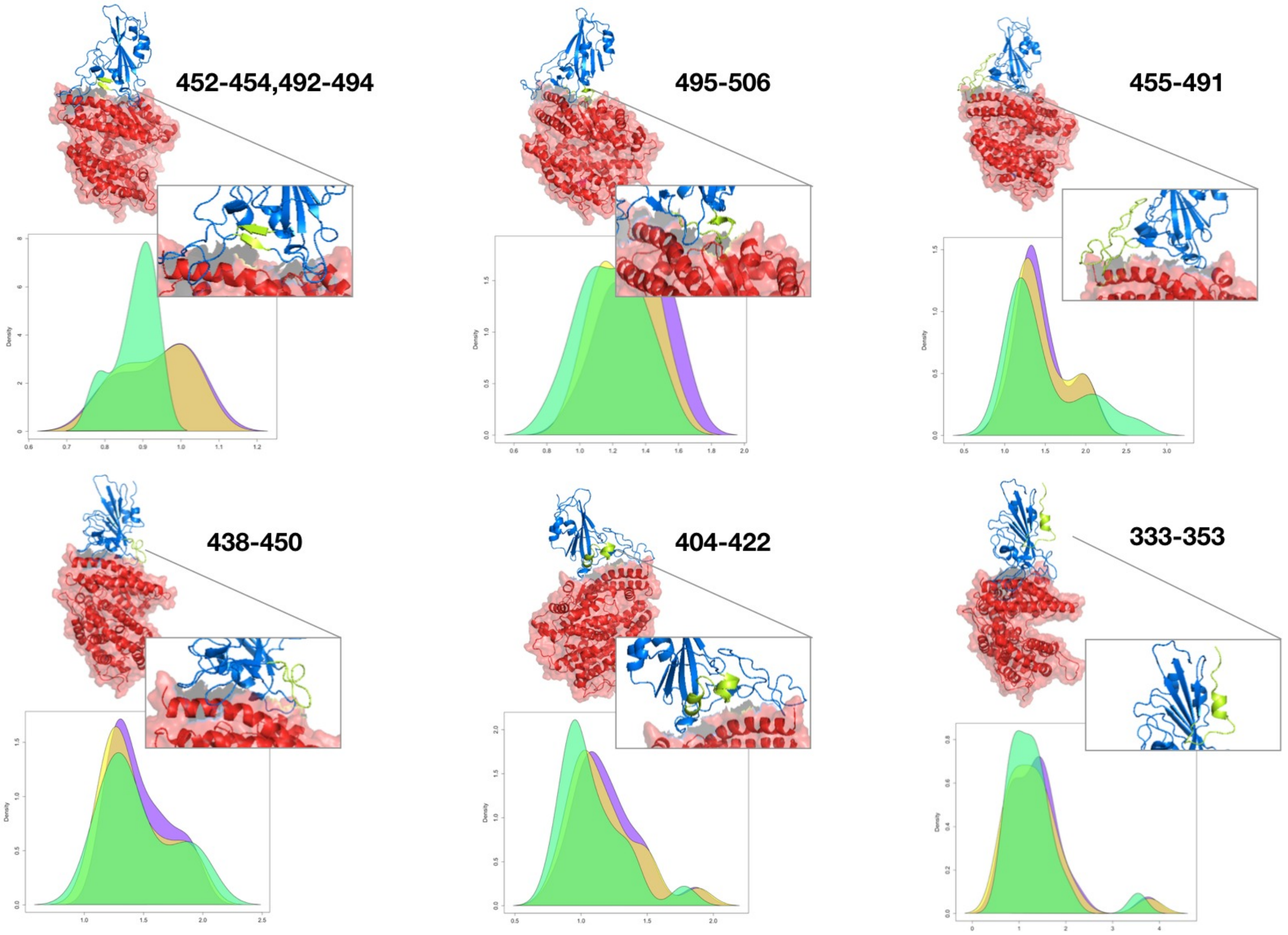

FIG. 11: **Fluctuations of Spike protein secondary structure residues.** Density distribution of the Root Mean Square Fluctuation (RMSF) of the residues forming the secondary structures of SARS-CoV-2 spike RBD in complex with the human ACE2 receptor. Cartoon representation of the complex with a zoom on the considered secondary structure is reported above the distribution panel for each considered secondary structure. Green, yellow and purple shaded curves are given considering all residues of the single-mutation variants whose binding affinity is lower than -3 kcal/mol, between -3 kcal/mol and -0.05 kcal/mol, and higher than -0.05 kcal/mol, respectively.

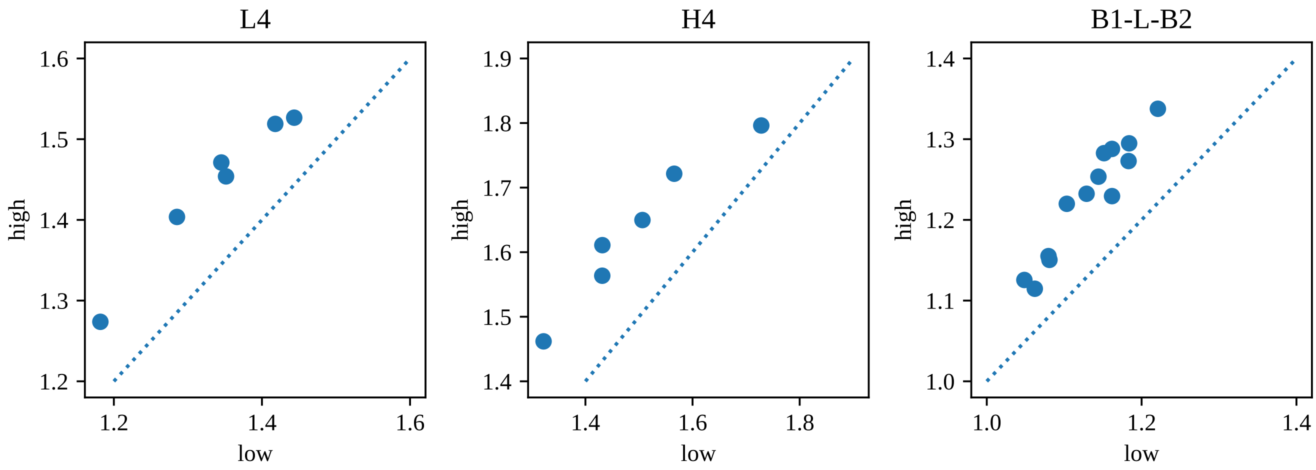

FIG. 12: **Mean RMSF values of the residues forming three structural motifs on human ACE2.** Root Mean Square Fluctuation (RMSF) of the residues forming the secondary structures of SARS-CoV-2 spike RBD in complex with the human ACE2 receptor. Averages are performed considering all residues of the single-mutation variants whose binding affinity is lower than -3 kcal/mol vs those higher than -0.05 kcal/mol.

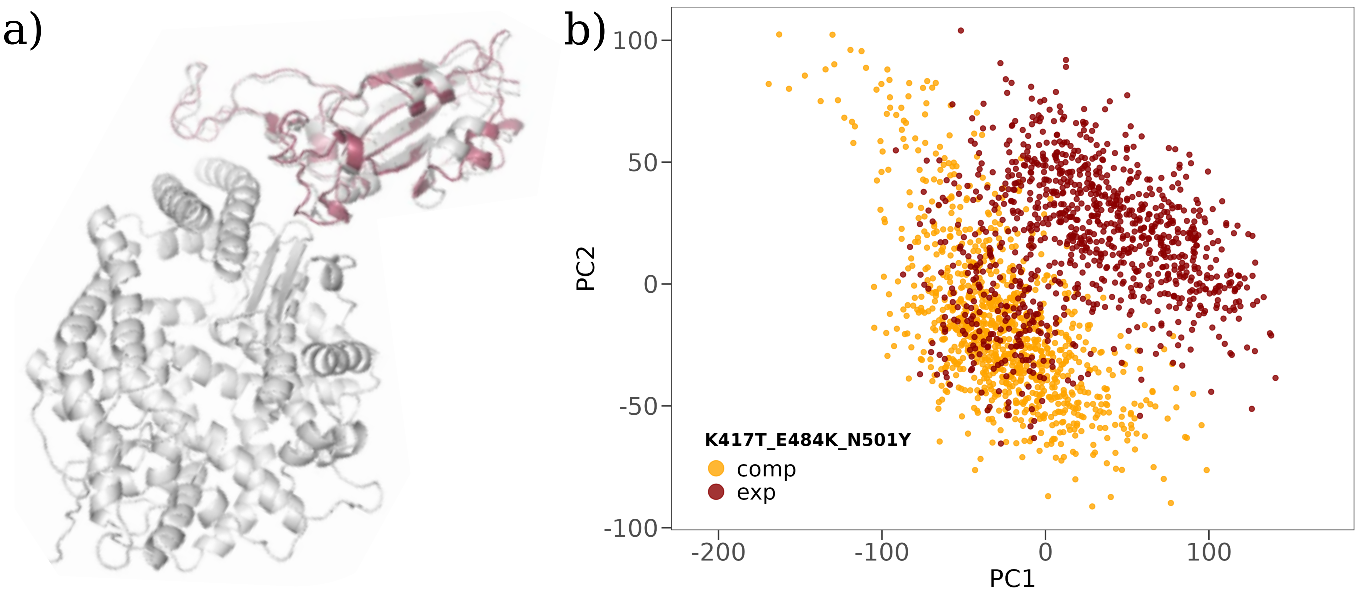

FIG. 13: **a)**Cartoon representation of two SARS-CoV2 spike-ACE2 complexes obtained from experimental data and molecular dynamics simulation. **b)**Comparison between the molecular dynamics simulation of the experimental and computationally-obtained complex of the gamma variant of SARS-CoV-2 spike protein bound to ACE2 receptor. Snapshots of the spike protein are projected in the plane of the two major components of the covariance matrix obtained from a principal component analysis (PCA) over the position covariance matrix.
